# A Unique Set of Auxiliary Metabolic Genes Found in an Isolated Cyanophage Sheds New Light on Marine Phage-Host Interactions

**DOI:** 10.1128/spectrum.02367-22

**Published:** 2022-10-03

**Authors:** Qiong Wang, Lanlan Cai, Rui Zhang, Shuzhen Wei, Fang Li, Yuanfang Liu, Yongle Xu

**Affiliations:** a Institute of Marine Science and Technology, Shandong University, Qingdao, People’s Republic of China; b State Key Laboratory of Marine Environmental Science, College of Ocean and Earth Sciences, Fujian Key Laboratory of Marine Carbon Sequestration, Xiamen University, Xiamen, People’s Republic of China; c Department of Ocean Science, The Hong Kong University of Science and Technology, Hong Kong, People’s Republic of China; d Southern Marine Science and Engineering Guangdong Laboratory (Zhuhai), Zhuhai, People’s Republic of China; e State Key Laboratory Breeding Base of Marine Genetic Resource, Third Institute of Oceanography, Ministry of Natural Resources, Xiamen, People’s Republic of China; University of Guelph

**Keywords:** auxiliary metabolic gene, cyanophages, phage-host interaction

## Abstract

Cyanophages, viruses that infect cyanobacteria, are abundant and widely distributed in aquatic ecosystems, playing important roles in regulating the abundance, activity, diversity, and evolution of cyanobacteria. A T4-like cyanophage, S-SCSM1, infecting *Synechococcus* and *Prochlorococcus* strains of different ecotypes, was isolated from the South China Sea in this study. For the first time, a mannose-6-phosphate isomerase (MPI) gene was identified in the cultured cyanophage. At least 11 phylogenetic clusters of cyanophage MPIs were retrieved and identified from the marine metagenomic data sets, indicating that cyanophage MPIs in the marine environment are extremely diverse. The existence of 24 genes encoding 2-oxoglutarate (2OG)-Fe(II) oxygenase superfamily proteins in the S-SCSM1 genome emphasizes their potential importance and diverse functions in reprogramming host metabolism during phage infection. Novel cell wall synthesis and modification genes found in the S-SCSM1 genome indicate that diverse phenotypic modifications imposed by phages on cyanobacterial hosts remain to be discovered. Two noncoding RNAs of *cis*-regulatory elements in the S-SCSM1 genome were predicted to be associated with host exopolysaccharide metabolism and photosynthesis. The isolation and genomic characterization of cyanophage S-SCSM1 provide more information on the genetic diversity of cyanophages and phage-host interactions in the marine environment.

**IMPORTANCE** Cyanophages play important ecological roles in aquatic ecosystems. Genomic and proteomic characterizations of the T4-like cyanophage S-SCSM1 indicate that novel and diverse viral genes and phage-host interactions in the marine environment remain unexplored. The first identified mannose-6-phosphate isomerase (MPI) gene from a cultured cyanophage was found in the S-SCSM1 genome, although MPIs were previously found in viral metagenomes at high frequencies similar to those of the cyanophage photosynthetic gene *psbA*. The presence of 24 genes encoding 2-oxoglutarate (2OG)-Fe(II) oxygenase superfamily proteins, novel cell wall synthesis and modification genes, a nonbleaching protein A gene, and 2 noncoding RNAs of *cis*-regulatory elements in the S-SCSM1 genome as well as the presence of a virion-associated regulatory protein indicate the diverse functions that cyanophages have in reprogramming the metabolism and modifying the phenotypes of hosts during infection.

## INTRODUCTION

The ocean contains abundant and enormously diverse viruses (1, 2), which induce approximately 20% of microbial mortality, influence microbial community composition and diversity, redirect metabolism, and impact the evolution of their hosts in the marine environment (3–5). Viral metagenomics studies have revealed that viruses harbor extremely high but largely uncharacterized genetic diversity (6). Furthermore, genes encoding proteins involved in various host metabolic pathways, known as auxiliary metabolic genes (AMGs), have been found frequently in viral genomes. Genomic and physiological characterizations of isolated viruses assist in interpreting unknown sequences found in viral metagenomes (7) and illustrating the phage-host interactions and ecological roles of viruses (8, 9).

*Prochlorococcus* and *Synechococcus* are the most abundant picocyanobacteria, contributing up to 50% of primary production in the marine environment (10, 11). Cyanophages exert significant influences on the ecological roles of picocyanobacteria (12). Approximately 1% to 5% of picocyanobacterial cells in the ocean are infected by cyanophages at one time (13–16). In the mid- to low-latitude ocean, cyanophages impact the population dynamics of picocyanobacteria, resulting in an average mortality rate of 0.14 day^−1^ (17). Cyanophages modify ecosystem-level productivity through the expression of AMGs, which include genes involved in photosynthetic electron transport and central carbon metabolism (18–22). The expression of these viral genes modifies host photosynthesis to maximize energy production and decrease carbon fixation during infection (23, 24). An estimated 0.02 to 5.39 Pg carbon per year is lost due to the virus-induced inhibition of CO_2_ fixation, which is important for estimations of global primary production (23). Cyanophages also harbor phosphorus acquisition genes (e.g., *pstS* and *phoA*), which assist in the uptake of phosphorus during phage infection under phosphorus-limited conditions (25). In addition, lipopolysaccharide (LPS) synthesis genes, including a NAD-dependent epimerase, a nucleotide-sugar epimerase, and a glycosyltransferase family 2 protein, have also been found in cyanophage genomes (17). The products of LPS genes, which modify host cell surface compositions (26), may affect host interactions and communications with other microbes in the marine environment, such as the attachment of other phages (26) and the recognition of grazers (27).

To date, all of the cyanophages isolated from the marine ecosystem belong to the order *Caudovirales*, which is currently divided into five families: *Myoviridae*, *Podoviridae*, *Siphoviridae*, *Ackermannviridae*, and *Herelleviridae* (28–31). Cyanomyoviruses are the most frequently isolated cyanophages in the aquatic environment (29). Most cyanomyovirus isolates are T4-like viruses that share a collection of core genes involved in the virion structure, DNA replication, and phage-host interactions (6, 18, 32). Various kinds of AMGs in T4-like cyanophage genomes are thought to drive diverse phage-host interactions (19, 33). In addition, noncoding RNA genes, including CFrI, PhotoRC-II, *wcaG*, and the glutamine riboswitch, are also present in T4-like cyanophage genomes and are predicted to modify the host’s metabolism (34–36). Here, we characterized a T4-like cyanomyovirus, S-SCSM1, which was isolated from the South China Sea and carries a new set of AMGs not found previously in other cyanophages. The isolation and characterization of S-SCSM1 could help further our understanding of phage-host interactions in the marine ecosystem.

## RESULTS AND DISCUSSION

### Isolation and biological characteristics of phage S-SCSM1.

**FIG 1:**
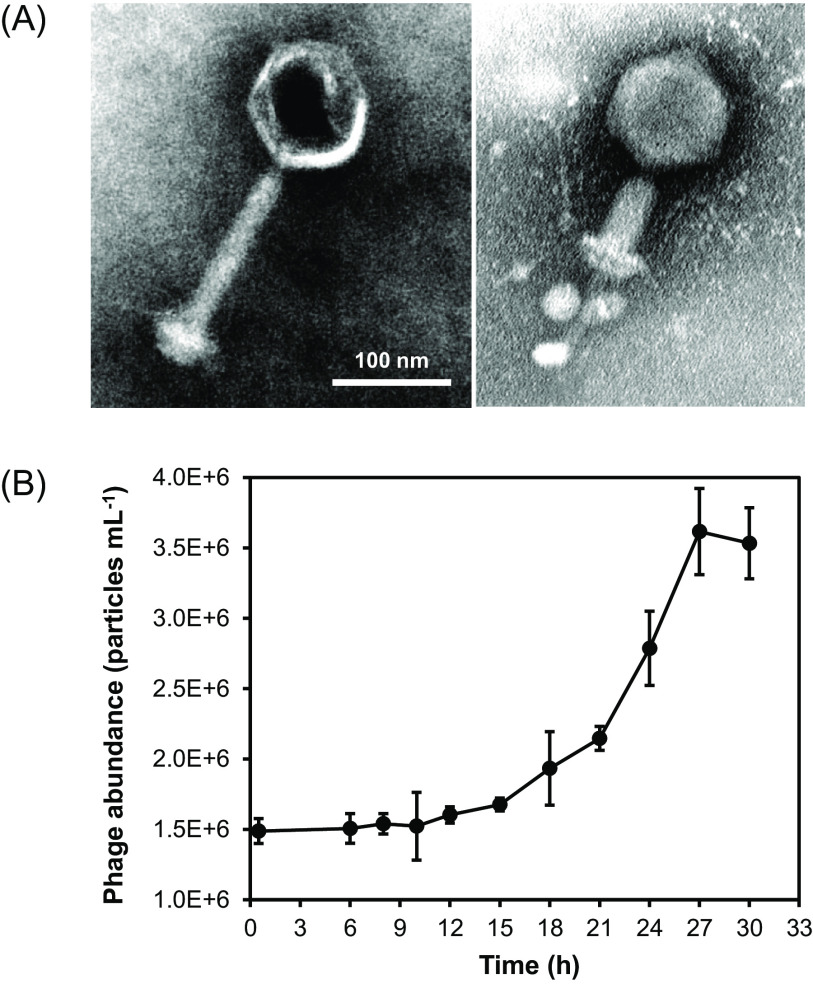
Biological characteristics of phage S-SCSM1. (A) Transmission electron microscopy images of cyanophage S-SCSM1 with a complete tail (left) and a contractile sheath (right). (B) One-step growth curve of cyanophage S-SCSM1.

Phage S-SCSM1 infecting *Synechococcus* sp. strain WH7803 was isolated from the surface seawater of the South China Sea (30.184°N, 7.726°E) in October 2013. Transmission electron microscopy observation reveals that S-SCSM1 is a myovirus with an icosahedral capsid (100 ± 1 nm in diameter) and a contractile tail (173 ± 4 nm long and 11 ± 1 nm wide) (Fig. 1A). The latent period of S-SCSM1 infecting *Synechococcus* sp. WH7803 was 15 to 18 h, and the first growth plateau occurred at 27 h, with a burst size of 50 (Fig. 1B). Consistent with previous findings that cyanomyoviruses usually exhibit broad host ranges (37), S-SCSM1 infects both *Synechococcus* and *Prochlorococcus* strains (Table 1), including *Synechococcus* sp. strains WH7803, WH7805, and WH8108, belonging to clades VI and V of subcluster 5.1, and *Prochlorococcus* sp. high light (HL) and low light (LL) strains MED4 (HLI), MIT9301 (HLII), AS9601 (HLII), MIT9313 (LLIV), and NATL2A (LLII). However, *Synechococcus* sp. strain CC9311, *Prochlorococcus* sp. strain NATL1A, and seven estuarine *Synechococcus* strains were resistant to infection by S-SCSM1 (Table 1). A recent study on marine cyanobacterial resistance to T4-like cyanophages with broad host ranges reveals that the defense is intracellular. In addition, the process of infection by the same phage in different cyanobacteria can be arrested at different stages, including phage genome transcription, translation, replication, and packaging (38). However, the resistance mechanisms remain unclarified since known intracellular defense mechanisms, such as restriction-modification and CRISPR-Cas, are absent from most marine *Synechococcus* and *Prochlorococcus* strains (38). Further studies on the interactions between S-SCSM1 and its resistant cyanobacterial strains may contribute to uncovering the marine cyanobacterial resistance mechanisms against cyanophages.

**TABLE 1:** Host range of phage S-SCSM1

Tested strain	Phylogenetic clade	Location of isolation (reference)	Result^*a*^
Marine strains			
*Synechococcus* sp. CC9311	I, subcluster 5.1	California current (108)	−
*Synechococcus* sp. WH7803	V, subcluster 5.1	Sargasso sea (109)	+
*Synechococcus* sp. WH7805	VI, subcluster 5.1	Sargasso sea (109)	+
*Synechococcus* sp. WH8108	VI, subcluster 5.1	Woods hole (109)	+
*Prochlorococcus* sp. MED4	HLI	Mediterranean sea (110)	+
*Prochlorococcus* sp. MIT9301	HLII	Sargasso sea (111)	+
*Prochlorococcus* sp. AS 9601	HLII	Arabian sea (112)	+
*Prochlorococcus* sp. NATL 1A	LLI	North atlantic (113)	−
*Prochlorococcus* sp. NATL 2A	LLII	North atlantic (114)	+
*Prochlorococcus* sp. MIT9313	LLIV	Gulf stream (115)	+
Estuarine strains			
*Synechococcus* sp. CB0101	CB4	Chesapeake bay (116)	−
*Synechococcus* sp. CBW1002	Bornholm sea	Chesapeake bay (117)	−
*Synechococcus* sp. CBW1004	Unclassified	Chesapeake bay (117)	−
*Synechococcus* sp. CBW1107	Subalpine C II	Chesapeake bay (117)	−
*Synechococcus* sp. CBW1001	Bornholm sea	Chesapeake bay (117)	−
*Synechococcus* sp. CBW1006	Subalpine C II	Chesapeake bay (117)	−
*Synechococcus* sp. CBW1101	Bornholm sea	Chesapeake bay (117)	−

a +, infected; −, uninfected.

### Genomic features of S-SCSM1.

**FIG 2:**
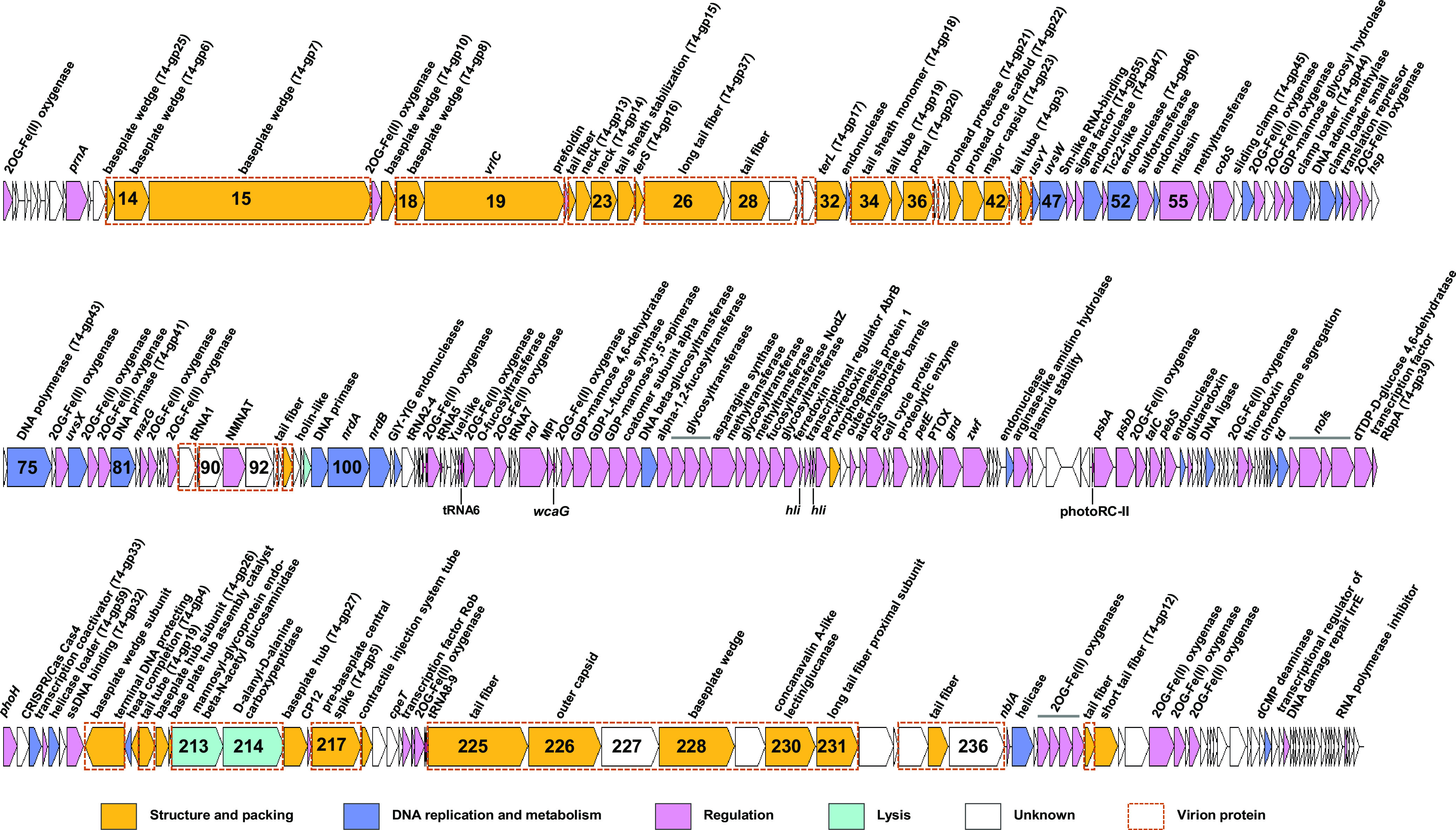
Genomic organization of S-SCSM1. Genes with different functions are indicated by different colors. The noncoding RNAs are annotated underneath the ORF bar. Virion proteins identified by mass spectrometry analysis are indicated by red dashed frames. 2OG, 2-oxoglutarate; NMNAT, nicotinamide/nicotinate mononucleotide adenylyltransferase; GIY-YIG, GlyIleTyr–TyrIleGly; MPI, mannose-6-phosphate isomerase; PTOX, plastoquinol terminal oxidase; ssDNA, single-stranded DNA.

The genome sequence of S-SCSM1 was assembled into a circularly permuted, double-stranded DNA molecule with a length of 228,827 bp and a G+C content of 36.8%. Totals of 282 open reading frames (ORFs), 9 tRNA genes, and 2 noncoding RNA genes were predicted in the S-SCSM1 genome (Fig. 2; see also Table S1 in the supplemental material). Only 163 ORFs had predictable functions. The other 119 ORFs had unknown functions, with 24 having no homologs in the GenBank nonredundant (NR) database. In addition, 228 ORFs of S-SCSM1 showed homology with those of T4-like cyanophages infecting either *Prochlorococcus* or *Synechococcus*, which indicates that S-SCSM1 is a member of the T4-like cyanophages (Table S1). S-SCSM1 shares the largest number (171) of ORF homologs with cyanophage S-SM2, which was isolated from *Synechococcus* sp. strain WH8017 from the Atlantic Ocean (18). The 163 ORFs with predictable functions in the S-SCSM1 genome were divided into three categories, genes related to structure formation, DNA replication and metabolism, and AMGs (Fig. 2; Tables S1 and S2).

All of the genes related to structure and DNA repair, replication, and metabolism in the S-SCSM1 genome have remarkable levels of synteny with those of T4-like cyanophages. In total, 37 ORFs in the S-SCSM1 genome were predicted to encode proteins related to structural formation, 33 of which shared amino acid sequence identities ranging from 27% to 83% with those of T4-like cyanophages. These structural genes were divided mainly into genes encoding head and tail proteins, which are universal among T4-like cyanophages. In total, 28 ORFs in the S-SCSM1 genome encode proteins involved in DNA replication, metabolism, and repair, and they share amino acid identities ranging from 30.7% to 94% with those of T4-like cyanophages. In addition to the AMGs related to photosynthesis, carbon metabolism, and phosphorus acquisition, which are prevalent in cyanophages (18), the S-SCSM1 genome contains genes related to cell wall synthesis and modification, nitrogen transformation, and plasmid stability maintenance (Fig. 2; Tables S1 and S2). Phylogenomic analysis of S-SCSM1 and 36 T4-like cyanophages based on 34 core genes showed that S-SCSM1 was most closely related to *Synechococcus* phage S-SM2 and fell into cluster A of the T4-like cyanophages as classified previously by Ignacio-Espinoza and Sullivan (6) (Fig. S1).

### First mannose-6-phosphate isomerase (MPI) gene found in a cyanophage isolate.

In addition to the genes involved in the pentose phosphate pathway (PPP), the S-SCSM1 genome also encodes an MPI (ORF118) (GenBank accession number QFG06376.1) involved in carbon metabolism. MPI catalyzes the interconversion of fructose-6-phosphate (F6P) and mannose-6-phosphate, which have important roles in mannose catabolism and glycolysis-, PPP-, and Calvin cycle-associated pathways (39). The phage may regulate the host’s central metabolism by influencing the F6P concentration in host cells during infection. In addition, MPI may allow the phage to use mannose for productions of deoxynucleoside triphosphate and reducing power under low-nutrient conditions because of the status of F6P in the PPP (39). Although MPIs were previously found in viral metagenomes at high frequencies similar to those of the cyanophage photosynthetic gene *psbA* (40), this is the first report of an MPI gene in the genome of an isolated cyanophage.

The MPI protein family contains four distinct classes (41, 42). The type I and III classes are monofunctional enzymes that have only mannose-6-phosphate isomerase activity. Members of the type II class are generally bifunctional proteins with both mannose-6-phosphate isomerase and GDP-d-mannose pyrophosphorylase activities in separate catalytic domains. However, the type II MPIs encoded in marine *Synechococcus* and *Prochlorococcus* genomes contain two groups, with one being the typical bifunctional enzyme possessing a mannose-6-phosphate isomerase and a GDP-d-mannose pyrophosphorylase domain and the other having only the mannose-6-phosphate isomerase domain. The type IV class proteins are also bifunctional, with both mannose-6-phosphate isomerase and glucose-6-phosphate isomerase activities (41, 42). A phylogenetic analysis based on MPIs of different organisms revealed that the S-SCSM1 MPI clustered within type II MPIs, which include those of cyanobacteria, bacteria, archaea, and chloroviruses (Fig. S2). The S-SCSM1 MPI is also a monofunctional enzyme, containing only the mannose-6-phosphate isomerase domain.

**FIG 3:**
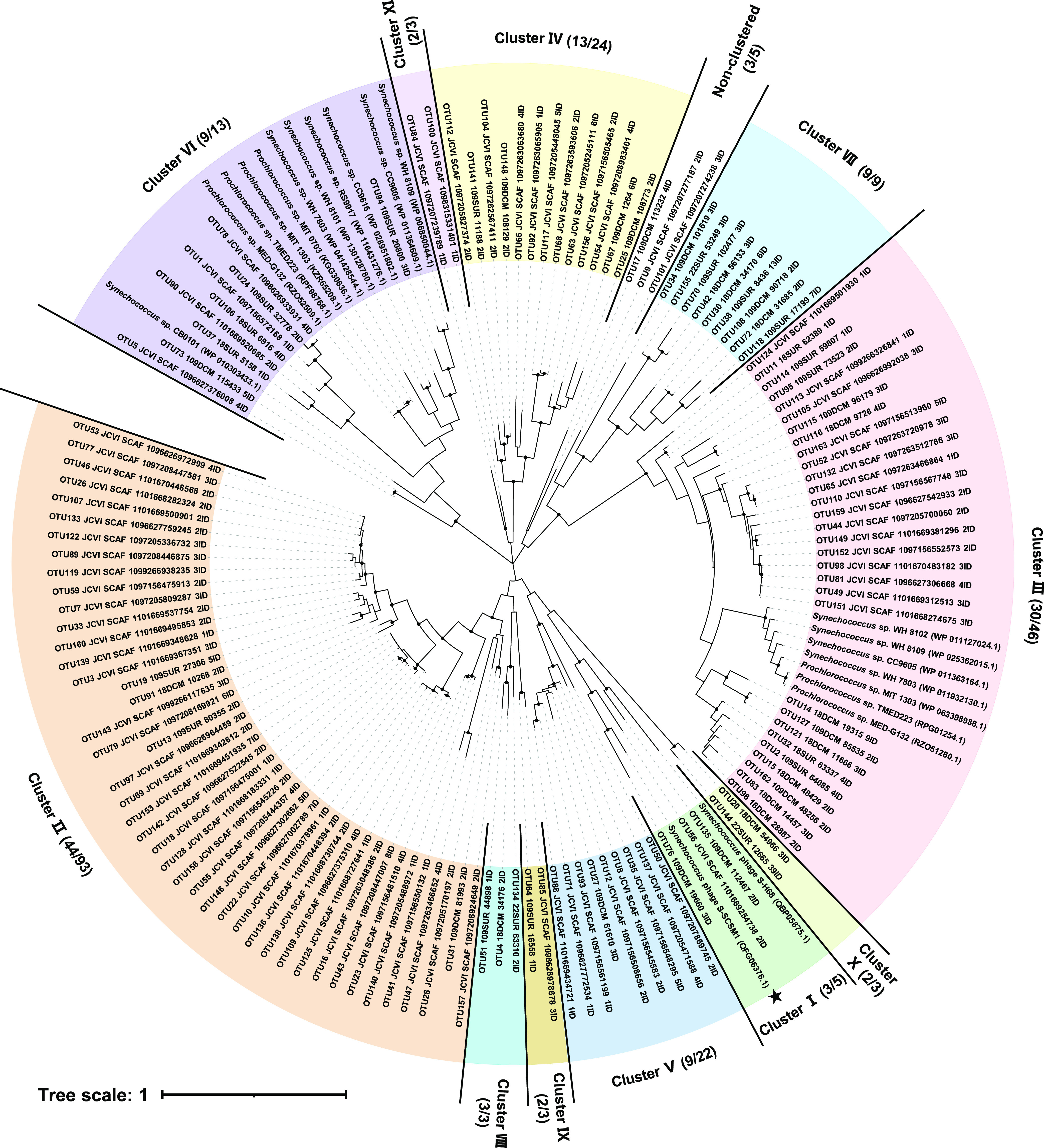
Unrooted maximum likelihood phylogenetic tree based on MPI amino acid sequences of cyanophages S-SCSM1 and S-H68, cyanobacteria, and environmental OTUs of cyanophage origin. MPI-containing environmental sequences of cyanophage origin were retrieved from the assembled *Tara* Oceans Viromes (TOV) and Global Ocean Sampling (GOS) databases. Nonclustered OTUs with amino acid sequence identities of >40% with the S-SCSM1 MPI were included in this phylogenetic analysis. The numbers of OTUs/sequences for each cluster are included in parentheses. Bootstrap values of ≥50% are indicated by filled circles. The number of bootstrap replicates was 1,000.

The evolution of viral MPIs remains to be addressed. MPIs have been found previously in *Bacillus* phages and chloroviruses. Phylogenetic analyses revealed that *Bacillus* phage MPIs are closely related to those retrieved from *Bacillus* strains and form a distinct type I lineage, which indicates that *Bacillus* phages may obtain MPIs from their hosts. However, chlorovirus MPIs, which belong to type II, are very different from those of their *Chlorella* hosts, which belong to type I (Fig. S2). To investigate the diversity and evolution of the cyanophage MPIs, 267 MPI sequences of cyanophage origin were retrieved from metagenomics databases using the S-SCSM1 MPI as the query. Like the S-SCSM1 MPI, the retrieved MPIs of cyanophage origin are all monofunctional enzymes with a mannose-6-phosphate isomerase domain. The environmental MPIs were grouped into 163 operational taxonomic units (OTUs) with 3% nucleotide sequence divergence, which indicated that cyanophage MPIs are highly diverse. The nonclustered OTUs showing amino acid sequence identities of <40% with the S-SCSM1 MPI were excluded to obtain a more topologically stable tree. In total, 129 OTUs, representing 229 sequences, were used to analyze the phylogeny of cyanophage MPIs and their evolutionary relationship with MPIs of cyanobacteria. Eleven clusters were identified in the phylogenetic analysis (Fig. 3). The S-SCSM1 MPI clustered with those of S-H68 and two environmental sequences and was defined as cluster I. Cluster II is the most abundant and diverse cluster, containing more than one-third (93 out of 229) of the total retrieved sequences. Of the 11 clusters, 2 are closely related to those of *Synechococcus* and *Prochlorococcus*. Members of clusters III and VI were grouped with the isomerase domain of the cyanobacterial bifunctional enzyme and the single isomerase, respectively, and they contained 22.1% of the total retrieved sequences, indicating that picocyanobacterial host MPIs are important origins from which cyanophage MPIs evolved. The other clusters diverged from those of S-SCSM1 and cyanobacteria and are distinct from each other, which indicates that cyanophage MPIs in the marine environment are extremely diverse and may have evolved from multiple origins.

**FIG 4:**
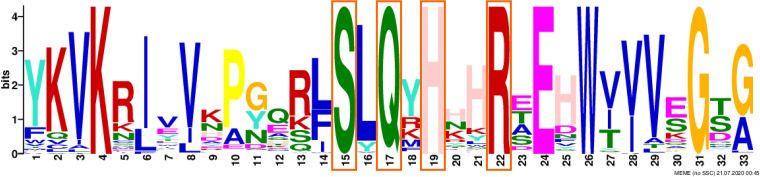
Motif 1 in the MPIs of representative cyanophage OTUs and picocyanobacterial amino acid reference sequences shown in Fig. 3. The functional sites Ser95, Gln97, His99, and R408 are indicated by red boxes. Letters are abbreviations of 20 different amino acids, and the letter size signifies the level of conservation at each site.

Although type II MPIs have been characterized in terms of function, MPI active sites, and important amino acid residues related to MPI activities in specific bacterial species (43–45), the above-described features and tertiary structures of MPIs in picocyanobacteria and cyanophages remain unexplored. Previous studies suggest that type I and II MPIs share a 9-residue motif that includes 3 residues (41), Ser95, Gln97, and His99, that are crucial for phosphate binding (46), catalysis and ring opening (47), and zinc binding and ring opening (48), respectively. Additionally, residue R408, which is in the 9-residue motif of type II MPIs, is important for MPI catalysis in Pseudomonas aeruginosa PAO1 (44). To evaluate whether MPIs in cyanophages are potentially functionally conserved, the amino acid sequences of each cluster were compared with those of well-characterized type I and II MPIs. The MPI representative OTUs and picocyanobacterial amino acid reference sequences in this study all contained the four important conserved residues in the same motif (Fig. 4), suggesting that they are functional at these specific sites. In addition, amino acid residues corresponding to the identified bacterial MPI active site are not conserved in all of the cyanophage and picocyanobacterial clusters, with only those of cluster III resembling the bacterial homologs (Fig. S3). However, the corresponding site within each cluster is conserved (Fig. S4). Furthermore, comparisons between the predicted tertiary structure of cluster I and those of the 10 other clusters identified in the phylogenetic analysis as well as that of the reference protein Pseudomonas aeruginosa PslB showed that they were similar at the MPI active site, with root mean square deviations of less than 3.000. Important conserved residues and diverse MPI active sites with conserved tertiary structure suggest that cyanophage MPIs of different clusters may function normally in a fashion similar to that of the previously identified bacterial homologs, but the MPI activities may differ among the clusters (Fig. S5).

### A large number of 2-oxoglutarate (2OG)-Fe(II) oxygenase superfamily genes.

Notably, the S-SCSM1 genome contains 24 ORFs encoding 2OG-Fe(II) oxygenase superfamily proteins (Fig. 2; Tables S1 and S2). 2OG-Fe(II) oxygenase superfamily proteins have predicted functions in many biological processes, such as protein modification, DNA repair, lipid metabolism, and secondary metabolite production, by catalyzing the hydroxylation of these molecules in microbes (49). The members of this superfamily have important effects on cell growth under nutrient-limiting conditions (49, 50). In addition, 2OG-Fe(II) oxygenases may provide a link between metabolism and transcriptional regulation in bacteria, which is thought to be crucial for regulating cell responses to environmental changes. Diverse 2OG-Fe(II) oxygenase genes are present in cyanophage and cyanobacterial genomes (51). Although most of the cultured T4-like cyanophage genomes have numerous 2OG-Fe(II) oxygenase genes, ranging from 1 to 5 (18), it is uncommon for a single phage genome to possess this large number of genes. The 24 2OG-Fe(II) oxygenase family genes were divided into four subfamilies on the basis of their most conserved domains, pfam13640, -13661, -13759, and -05721, and they show low amino acid sequence identities among themselves, with the highest being 44.2% (Table S3). In addition, the S-SCSM1 2OG-Fe(II) oxygenase family genes also show low amino acid sequence identities with those of other cyanophage isolates, ranging from 34.3% to 57% (Table S1). In phylogenetic analyses, the 24 genes formed at least 13 clusters (Fig. S6). ORF16, -71, -77, -79, -80, -107, -120, -170, -240, and -243 share the conserved pfam13759 domain. Most of the pfam13759 ORFs cluster with the cyanophage homologs, with ORF16 and -107 showing distant homology with proteins predicted in a filamentous cyanobacterium and a *Euryarchaeota* archaeon. ORF63, having a domain that was the most identical to pfam13661, formed an individual deep branch. ORF1, -61, -84, -88, -112, -224, -241, -242, -249, and -251, all of which possess a region that is the most identical to the conserved pfam13640 domain, formed at least seven clades with those from cyanophages. In addition, ORF88 and ORF249 also clustered with those of *Synechococcus* and *Prochlorococcus*, which indicates that they may share an ancestor (Fig. S6). A study by Ma et al. on the phylogeny of the 2OG-Fe(II) oxygenase superfamily proteins reveals that cyanophage genes cluster with those of other cyanophages rather than with cyanobacterial host sequences, and this suggests that cyanophage 2OG-Fe(II) genes may be exchanged mainly among the phage gene pool (51). However, the grouping of ORF88 and ORF249 with cyanobacteria reveals that 2OG-Fe(II) oxygenase gene exchange also occurs between cyanophages and cyanobacterial hosts. ORF114 and -186 contain a region that is the most identical to the conserved pfam05721 domain and formed two clusters with cyanophages and *Prochlorococcus* (Fig. S6). Notably, ORF248 contains two regions that are identical to the conserved domains pfam13640 and -05721 and shows homology with those of cyanophages (Fig. S6). At present, the functions of these genes in viruses have not been specifically investigated. Cyanophage 2OG-Fe(II) oxygenase is predicted to influence host nitrogen metabolism and energy production by regulating the cellular levels of 2-oxoglutarate during infection (18). The large number of diverse 2OG-Fe(II) oxygenase genes in the S-SCSM1 genome indicates their importance and various functions in reprogramming host metabolism during infection.

### Cell wall synthesis and modification genes.

The S-SCSM1 genome encodes 13 proteins that catalyze the production or transformation of important constituents of bacterial cell wall synthesis and modification (Fig. 2; Tables S1 and S2). GDP-mannose glycosyl hydrolase (ORF64) hydrolyzes GDP-mannose to GDP and mannose, providing mannosyl components for cell wall synthesis (52). GDP-mannose 4,6-dehydratase (ORF121) and GDP-l-fucose synthase (ORF122) catalyze the synthesis of GDP-l-fucose, which is the precursor of bacterial polysaccharides (53). GDP-mannose-3,5-epimerase (ORF123) catalyzes the conversion of GDP-mannose to GDP-l-galactose and GDP-l-gulose. Bacterial GDP-mannose-3,5-epimerases are predicted to play a role in the biosynthesis of l-galactose moiety-containing antibiotics and LPS (54). The GDP-fucose protein *O*-fucosyltransferase (ORF113), α-1,2-fucosyltransferase (ORF126), and the fucosyltransferase NodZ (ORF134) function in the fucosylation of glycoconjugates, including glycoproteins, lactosamine, chitin oligomers, and lipooligosaccharide, and play roles in glycan metabolism and antigen synthesis (55, 56). Glycosyltransferases (ORF127, -128, -129, -132, and -135) catalyze the transfer of sugar moieties from activated donor molecules to specific acceptor molecules, which may be a growing oligosaccharide, a lipid, or a protein, to synthesize polysaccharides and glycoconjugates, including cellulose and LPS, which are important constituents of the bacterial cell wall (57). dTDP-d-glucose 4,6-dehydratase (ORF198) catalyzes the second of four steps in the dTDP-l-rhamnose pathway in the synthesis of l-rhamnose, which is an important constituent of LPS O-antigen (58). LPS and other cell wall synthesis and modification genes have been identified previously in other T4-like cyanophages with large genomes, such as P-SSM2 (252 kb) (19) and S-SSM7 (232 kb). LPS synthesis genes are found in both temperate and lytic phages, and they may modify the host’s cell surface composition to prevent infection by other phages during the lysogenic or pseudolysogenic phase (19, 59). The expression of phage LPS and other cell wall synthesis and modification genes during infection may affect host phenotypes for phage attachment, recognition by grazers, and communications and interactions with other cells (26, 27, 60). Of the 13 S-SCSM1 cell wall-related genes, six (ORF113, -122, -127, -129, -132, and -134) were identified here for the first time in a cyanophage genome, indicating that the mechanisms used by phages to impose diverse phenotypic modifications on cyanobacterial hosts remain to be discovered.

### A nonbleaching protein A (NblA) gene.

The S-SCSM1 genome carries one gene encoding a homolog of NblA (ORF237) (GenBank accession number QFG06501.1). In cyanobacteria, NblA is essential for the degradation of phycobilisomes (PBSs), which is the major light-harvesting complex (61). *nblA* genes were previously found in the genomes of freshwater cyanophages such as Ma-LMM01, MaMV-DC, and PaV-LD (62–64) and marine viral metagenomes (65). Cyanophage *nblA* genes are expressed during infection, which was accompanied by significant declines in the host’s phycobilin content (62, 64, 66). The heterologous expression of freshwater cyanophage *nblA* genes in *Synechocystis* sp. strain PCC 6803 and *nblA* genes identified from marine viral metagenomes in Synechococcus elongatus PCC 7942 resulted in spectroscopic shifts associated with phycobilin loss, indicating that cyanophage NblA proteins also induce PBS degradation (64–66). The PBSs in cyanobacteria constitute up to 50% of the total cellular soluble proteins (67). The degradation of PBSs by the phage NblA protein may not only release nutrients in the form of amino acids, which can ease nitrogen starvation triggered by phage replication, but also prevent photodamage by reducing the absorption of excess light energy, which can maintain photosynthetic energy generation during infection (63, 68).

**FIG 5:**
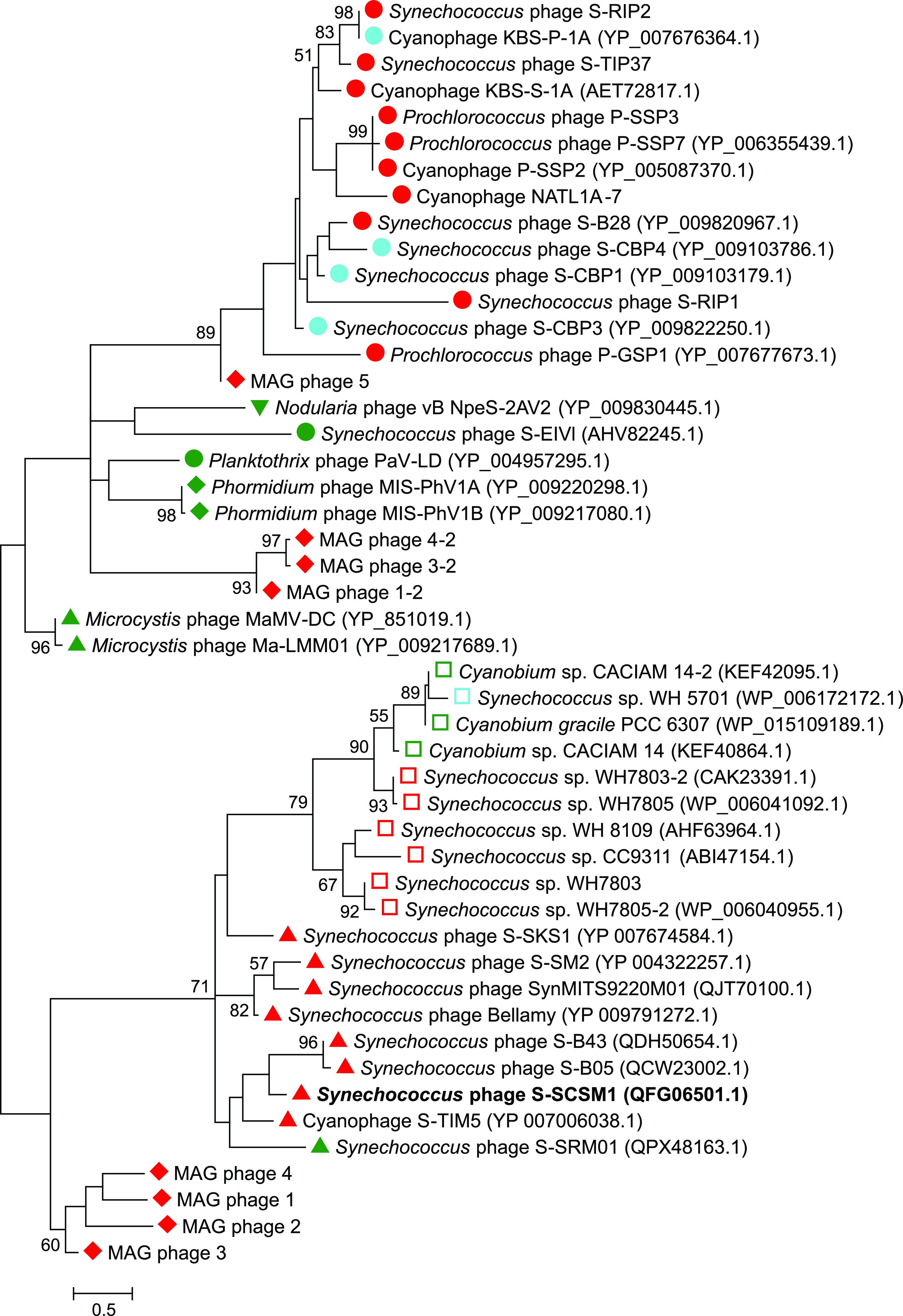
Unrooted maximum likelihood phylogenetic tree based on *nblA* amino acid sequences of cyanophages, picocyanobacteria, and environmental sequences of cyanophage origin. Except for sequences of six marine myoviruses that are homologous to S-SCSM1, other reference sequences were obtained from the data set reported previously by Nadel et al. (53). Sequences of the cyanopodovirus, cyanomyovirus, cyanosiphovirus, and unknown environmental types of cyanophage origin are indicated with circles, triangles, inverted triangles, and diamonds, respectively. Picocyanobacterial sequences are indicated by squares. The habitat types of marine water, brackish water, and freshwater are represented by red, cyan, and green, respectively. Bootstrap values of ≥50% are shown near each node. The number of bootstrap replicates was 1,000.

Besides *nblA* genes identified from the marine metagenome-assembled genomes (MAGs), totals of 11 marine and 4 estuarine cyanophage isolates, including 14 cyanopodovirus strains and 1 cyanomyovirus (S-TIM5) strain (Fig. 5), were also predicted to contain an *nblA* gene by Nadel et al. (65). The putative *nblA* gene of S-SCSM1 was predicted by distant protein homology detection using HHpred (69). In addition, by applying a BLASTP analysis of the S-SCSM1 *nblA* gene against the NR database, homologs from six marine and one freshwater cyanomyoviruses were also identified. These S-SCSM1 *nblA* homologs from cyanomyoviruses are also predicted to be distant homologs of cyanobacterial *nblA* genes by an HHpred search (Table S4). Phylogenetic analyses revealed that *nblA* genes of marine and estuarine cyanophages are distant from those of the previously identified freshwater cyanophages (Fig. 5). The marine and estuarine cyanopodovirus *nblA* genes grouped into a cluster with a MAG phage sequence (Fig. 5). The S-SCSM1 *nblA* genes and its seven cyanomyovirus homologs clustered with the S-TIM5 and picocyanobacterial *nblA* genes (Fig. 5), indicating that these cyanophage *nblA* genes may originate from their hosts or that *nblA* exchange occurred between these cyanophages and their hosts.

### A plasmid-related ORF.

Interestingly, S-SCSM1 ORF163 encodes a plasmid stability protein (GenBank accession number QFG06424.1). Genes encoding plasmid stability proteins are frequently found in cyanophage genomes. The viral plasmid stability protein functions in gene cassette mobility (34). S-SCSM1’s plasmid stability protein may have a similar function and contribute to horizontal gene transfer between the phage and the host during infection. Moreover, cyanophages can enter a pseudolysogenic state, in which the genome remains within the host cell like a plasmid, when infecting hosts under phosphate-depleted conditions (29). It is possible that the plasmid-related genes in cyanophage genomes function to maintain the pseudolysogenic state when an infection occurs under conditions unfavorable to phage progeny production.

### Noncoding RNAs are prevalent in cyanophage genomes.

Noncoding RNAs are important functional RNAs that do not encode proteins (70). They perform many functions that are essential for protein synthesis, RNA processing, and gene regulation (34). Noncoding RNAs are prevalent and well characterized in bacteria and archaea (34). However, there are limited studies on phage noncoding RNAs. An antisense RNA, CFrI, was previously identified in the genome of cyanophage S-PM2 and a set of metagenomic sequences of cyanophage origin. CFrI is complementary to the 3′ end of the cyanophage *psbA* gene and is predicted to regulate the expression of *psbA* during infection (36). *cis*-regulatory RNAs were previously identified in the untranslated regions of T4-like cyanophages, as indicated in the Rfam database (Table S5). Two *cis*-regulatory RNA genes, PhotoRC-II and *wcaG*, were predicted in the S-SCSM1 genome. *cis*-regulatory elements act as detectors of changes in environmental conditions, such as light or temperature variations, and regulate message stability or translational efficiency (34, 35). PhotoRC-II RNA has been detected in the genomes of 15 marine cyanophages (Table S5) and metagenomes (34). PhotoRC-II RNA is usually located upstream of *psbA* and acts as a PhotoRC-II motif to regulate the photosystem. It is possible that S-SCSM1’s PhotoRC-II RNA upstream of *psbA* regulates host photosynthesis during infection. The *wcaG* RNA typically appears to regulate genes related to exopolysaccharide production or those induced under high-light conditions. The *wcaG* RNAs may be employed by phages to modify host metabolism to adapt to environmental conditions during infection (71).

### Virion-associated proteins.

In total, 45 S-SCSM1-encoded proteins were detected in the virion proteome by mass spectrometry analysis. Of the 45 phage proteins, 29 were related to viral structure, including portal, major capsid, and tail proteins (Fig. 2). Of the remaining 16 proteins, 13 have unknown functions, with 2 having no matches in the NR database. Three proteins, nicotinamide/nicotinate mononucleotide (NMN) adenylyltransferase (NMNAT) (ORF91) (GenBank accession number QFG06348.1), mannosyl-glycoprotein endo-β-*N*-acetylglucosaminidase (ORF213) (accession number QFG06477.1), and d-alanyl-d-alanine carboxypeptidase (ORF214) (accession number QFG06478.1) are not likely to have structural functions. These proteins may be carried by the virion or highly expressed during phage infection, and they may have remained in the virion suspension after CsCl purification. NMNAT catalyzes the conversion of NMN to NAD and plays an important role in the biosynthesis of NAD (72). Beyond its role in redox reactions, NAD acts in reactions regulating fundamental cellular metabolism, including DNA ligation, ADP-ribosylation, and protein deacetylation (73–75). The maintenance of proper NAD levels is important for living cells (76). If the S-SCSM1 NMNAT is carried by the virion and could enter the host cell during infection, it may regulate host metabolism by influencing the NAD levels to facilitate phage progeny production. Notably, an NMNAT was previously detected in the virion proteome of an estuarine *Synechococcus* phage, S-CBWM1 (77). In addition, a predicted cytidylyltransferase that shows 39.8% amino acid identity with the S-SCSM1 NMNAT was also detected in the virion proteome of an estuarine *Synechococcus* phage (78). The frequent detection of NMNAT-like proteins in cyanophage virion proteomes indicates the high possibility that NMNAT-like proteins are carried by the virions and are essential for phage infection. Mannosyl-glycoprotein endo-β-*N*-acetylglucosaminidase may function as a lytic enzyme to destroy host peptidoglycan, whereas d-alanyl-d-alanine carboxypeptidase is thought to function as an endolysin to break the cell wall barrier (79). Mannosyl-glycoprotein endo-β-*N*-acetylglucosaminidase and d-alanyl-d-alanine carboxypeptidase may play roles in the phage’s entry into, or release from, host cells.

### Conclusion.

Cyanophage S-SCSM1, infecting both *Synechococcus* and *Prochlorococcus*, contains a set of conserved genes related to viral structure, DNA replication, and metabolism; a unique set of AMGs; and two noncoding RNAs of *cis*-regulatory elements. The presence of unique AMGs and noncoding RNAs may provide advantages to phages in harsh environments and act as potential gene pools for shaping their hosts’ evolution (80). The first discovered MPI gene, *nblA*, and a large number of diverse 2OG-Fe(II) oxygenase genes were found in the S-SCSM1 genome, providing new insights into the evolution of cyanophage AMGs. A collection of novel cell wall synthesis and modification genes and two noncoding RNA genes shed new light on phage-host interactions. The source and function of plasmid genes in viral genomes are still puzzling. In addition, the S-SCSM1 virion may encapsulate or highly express a phage regulatory protein. This study highlights the versatility of cyanophages, indicating that more efforts are needed to isolate cyanophages from various environments. Additionally, the characterization of novel cyanophages will reveal the vast viral diversity and illustrate the ecological roles of viruses.

## MATERIALS AND METHODS

### Phage isolation.

As one of the most sensitive and widely used marine *Synechococcus* strains (21), *Synechococcus* sp. WH7803 was used as the host cyanobacterium for phage isolation. It was cultured in A^+^ medium (81) at 22°C under cool-white light at an intensity of 20 μE m^−2^ s^−1^. Surface seawater from the South China Sea (30.184°N, 7.726°E) was filtered through a 0.22-μm-pore-size polycarbonate membrane filter (Millipore, Bedford, MA, USA) and stored at 4°C until use. S-SCSM1 was isolated by adding 20 μL of the seawater filtrate to 180 μL of exponentially growing WH7803 cultures (optical density at 750 nm [OD_750_] = 0.5) in a 96-well microtiter plate, while WH7803 cultures receiving 20 μL of A^+^ medium in triplicates were used as the controls. After cell lysis, the lysates were collected by centrifugation at 10,000 × *g* for 10 min and filtration through a 0.22-μm filter to remove cyanobacterial cells. Phage purification was carried out using the plaque assay method described previously by Suttle and Chen (82), three times.

### Host range.

Picocyanobacterial strains used in the host range test included *Prochlorococcus* sp. strains MED4, MIT9301, MIT9313, AS9601, NATL1A, and NATL2A; *Synechococcus* marine strains WH7803, WH7805, CC9311, and WH8108; and *Synechococcus* estuarine strains CB0101, CBW1002, CBW1004, CBW1107, CBW1001, CBW1006, and CBW1101. Phage suspensions were added to exponentially growing cultures of cyanobacterial strains in 96-well microtiter plates in triplicate and incubated for 1 to 2 weeks under the same conditions as the ones described above. Cultures were monitored daily for cell lysis.

### Infection dynamics.

A one-step growth experiment was performed to characterize the life cycle of phage S-SCSM1 using methods described previously (83). As the original host for S-SCSM1 isolation, *Synechococcus* sp. WH7803 was used as the host for the infection dynamics analysis. Phages were added to exponentially growing *Synechococcus* sp. WH7803 cultures in triplicate at a multiplicity of infection (MOI) of 0.1 and incubated for 1 h to allow phage adsorption to the host cells. Unabsorbed phage particles were removed by centrifugation. The resuspended cultures were then incubated at 22°C under cool-white light at an intensity of 20 μE m^−2^ s^−1^. Subsamples were taken for 30 h at 3-h intervals to monitor the variations in the viral concentration using a flow cytometer (Epics Altra II; Beckman Coulter, Miami, FL, USA) according to methods described previously (84, 85). Phage-forming units at the beginning of the incubation after phage adsorption and the first maximum release of viral particles were determined to calculate the burst size using a plaque assay method described previously (86).

### Phage amplification and purification.

Phage suspensions were added to 2 L of exponentially growing host cultures (OD_750_ = 0.5) at an MOI of 0.1 for phage amplification. The lysates were treated with 4 mg DNase І and 4 mg RNase A for 1 h to remove host DNA and RNA, respectively. After digestion, the NaCl concentrations in the lysates were adjusted to 1 M, and the mixtures were incubated at 4°C for 1 h (87). The treated lysates were centrifuged at 10,000 × *g* at 4°C for 20 min in a Beckman AR centrifuge. The supernatants were collected and filtered through 0.22-μm filters to remove the remaining cell debris. The phage particles in the supernatants were concentrated by tangential flow filtration with a 30-kDa polysulfone cartridge (Labscale; Millipore, Temecula, CA, USA) and then purified by CsCl gradient centrifugation (200,000 × *g* at 4°C for 8 h). The visible phage band was extracted. Centrifugal ultrafiltration was used to remove the CsCl from the phage suspension.

### Transmission electron microscopy observations.

After purification and desalting, 10-μL phage suspensions were adsorbed to a 200-mesh carbon-copper film for 20 min in the dark. Next, the sample was stained with 1% phosphotungstic acid for 10 min. The stained sample was dried for 2 h and then observed at 120 kV using a JEM-2100 transmission electron microscope (JEOL, Tokyo, Japan). The Gatan Inc. (Pleasanton, CA, USA) charge-coupled device (CCD) image transmission system was used to capture images.

### DNA extraction and genome sequencing.

CsCl-purified phage particles were treated with proteinase K (100 mg mL^−1^), SDS (10%, wt/vol), and EDTA (0.5 mol L^−1^) (pH 8.0) and incubated at 55°C for 3 h. Afterward, the phage DNA was extracted using a phenol-chloroform method as described previously by Chen et al. (88). A whole-genome shotgun strategy was used to construct the paired-end 400-bp (PE400) library. Genome sequencing was conducted on an Illumina MiSeq platform. The adapters were removed using AdapterRemoval (version 1.5.4). All of the reads were adjusted using Quake (version 0.3) and then assembled using A5-miseq (version 20150522). Gaps were filled using GapCloser (https://sourceforge.net/projects/soapdenovo2/files/GapCloser).

### Genomic and phylogenetic analyses.

The ORFs of the phage genome were predicted using the GeneMark.hmm 2.0 gene prediction program (89), the RAST server (http://rast.nmpdr.org/), and MetaGeneAnnotator (http://metagene.nig.ac.jp/). Translated ORFs were annotated by a BLASTP search against the NCBI NR database with an E value cutoff of <10^−5^ (90). A conserved domain search against the NCBI Conserved Domain Database was used to aid in the functional prediction of each ORF (91). For ORFs with no predicted functions based on sequence analyses, HHpred and Phyre2 analyses were conducted using default parameters to predict protein functions based on predicted structural properties (69, 92). An ORF homolog search was conducted between cyanophage S-SCSM1 and Escherichia phage T4 (GenBank accession number AF158101) with an E value cutoff of <10^−5^ to identify classic T4-like genes in the S-SCSM1 genome. The tRNAscan-SE program was used to identify tRNA sequences in the phage genome (93). The genome sequence was searched against the Rfam database to identify noncoding RNAs (35). Phylogenomic analyses were conducted among S-SCSM1 and 36 T4-like cyanophages based on the amino acid sequences of the core genes. Conserved single-copy core genes among the 37 cyanophage genomes were identified by OrthoFinder (94). Sequences were aligned using MAFFT (95) and trimmed using TrimAI (96). The phylogenomic tree was constructed with RAxML version 8 (97) using the maximum likelihood method with the PROTGAMMAJTT model (bootstrap replicates = 100). Phylogenetic trees of specific genes were constructed using the MEGA 7.0 software package (98). The maximum likelihood method based on the Jones-Taylor-Thornton (JTT) model and the neighbor-joining method based on the *p*-distance model with 1,000 bootstrap replicates were used for phylogenetic tree construction. The MPI phylogenetic tree of cyanophage origin was visualized using iTOL (99).

### Recruitment of the S-SCSM1 MPI homologs from metagenomic data sets.

S-SCSM1 MPI homologs were retrieved from the assembled *Tara* Oceans Virome (TOV) and Global Ocean Sampling (GOS) data sets (100). Both data sets were downloaded from the iMicrobe website (https://www.imicrobe.us/). The amino acid sequence of the S-SCSM1 MPI was used as a query to search against the two assembled metagenomic databases. Homologous sequences from data sets were identified using TBLASTN, with a threshold E value of 10^−5^, a bit score of >40, and a minimum amino acid length of 30, and extracted as previously described (101). Next, the retrieved scaffold sequences were screened for cyanophage MPIs using TBLASTX analysis against the NR database. MPIs and their adjacent genes in scaffolds having the best hits that belonged to cyanophages were identified as being of cyanophage origin. MPI sequences of cyanophage origin retrieved from metagenomes were grouped into OTUs with 97% nucleotide sequence identities using QIIME (102).

### MPI protein structural analyses.

MPI motifs were predicted by Multiple Em for Motif Elicitation (http://meme-suite.org/tools/meme) (103). Amino acid sequences of cyanophage and picocyanobacterial MPIs were aligned using MEGA 7.0 (104). Protein tertiary structures were predicted and compared using the I-TASSER (https://zhanglab.ccmb.med.umich.edu/I-TASSER/) (105) and PyMOL (106) software packages, respectively.

### Determination of virion proteins by mass spectrometry and identification of the corresponding genes in the genome.

CsCl-purified phage suspensions were used for the proteomics analysis of S-SCSM1. Viral proteins were digested using the filter-aided sample preparation procedure (107). The phage suspension was dissolved in 50 μL of SDT lysis buffer (4% SDS, 100 mM dithiothreitol [DTT], 150 mM Tris-HCl [pH 8.0]) for 10 min at 99°C. Next, 100 μL of UA buffer (8 M urea, 150 mM Tris-HCl [pH 8.0]) was used to remove the detergent from the protein solution through ultrafiltration. Afterward, 100 μL of 0.05 M iodoacetamide was used to modify the UA-buffered sample for 20 min at 25°C in the dark. The protein suspension was washed with 100 μL of UA buffer three times and then with 100 μL of 25 mM NH_4_HCO_3_ twice and then digested with 2 μg of trypsin (Promega) in 40 μL of 25 mM NH_4_HCO_3_ overnight at 37°C. The tryptic peptides were analyzed using a Q Exactive mass spectrometer (Thermo Fisher Scientific, Waltham, MA, USA) that was connected to an Easy nLC 1000 system (Thermo Fisher Scientific) (107). Next, a C_18_ reversed-phase analytical column (75 μm by 25 cm; Thermo, USA) was used to carry out peptide fractionation with buffer A (2% acetonitrile and 0.1% aqueous formic acid) and buffer B (80% acetonitrile and 0.1% aqueous formic acid) at a flow rate of 300 nL min^−1^. The mass spectrometry data were analyzed using Mascot 2.2 software (Matrix Science, London, UK). A total of 974 peptides obtained from the determination of virion proteins were searched against the amino acid sequences of S-SCSM1 ORFs to identify the corresponding genes in the genome.

### Data availability.

The genome sequence of *Synechococcus* phage S-SCSM1 has been submitted to the GenBank database under accession number MK867354.2.
