# Supplementary material for: A Unique Set of Auxiliary Metabolic Genes Found in an Isolated Cyanophage Sheds New Light on Marine Phage-Host Interactions
Source: Microbiol Spectr. 2022 Oct 3;10(5):e02367-22. doi: 10.1128/spectrum.02367-22 (PMC9602691; doi:10.1128/spectrum.02367-22)
Supplement: Supplemental file 1 — Tables S1 to S5 and Fig. S1 to S6. Download spectrum.02367-22-s0001.pdf, PDF file, 1.6 MB [file spectrum.02367-22-s0001.pdf]

Table S1. Predicted ORFs in the S-SCSM1 genome that have homologs in the GenBank non-redundant database.

| Gene | Strand | Left | Right | aa length | Significant hits in NR database[a] | Putative function[b] | E-value | aa identity | Conserved domain (e-value)[c] |
|---|---|---|---|---|---|---|---|---|---|
| 1 | + | 1 | 531 | 176 | hypothetical protein SSSM7_036 [*Synechococcus* phage S-SSM7] | | 1.00E-30 | 36% | |
| 2 | + | 531 | 749 | 72 | hypothetical protein [*Synechococcus* phage S-H34] | | 2.00E-19 | 64.2% | |
| | | | | | rnf gene product [*Synechococcus* phage S-CBS4] | | 1.00E-16 | 57% | |
| 3 | + | 685 | 852 | 55 | hypothetical protein S050808_056 [*Synechococcus* phage S-CAM9] | | 2.00E-07 | 54% | |
| 4 | + | 1207 | 1374 | 55 | hypothetical protein [uncultured Mediterranean phage uvMED] | | 2.00E-17 | 64% | |
| | | | | | hypothetical protein SSM2_088 [*Synechococcus* phage S-SM2] | | 9.12E-14 | 65.2% | |
| 5 | + | 1515 | 1862 | 115 | hypothetical protein C350210_066 [*Synechococcus* phage S-CAM22] | | 8.00E-62 | 80% | |
| 6 | + | 1927 | 2082 | 51 | hypothetical protein PBI_BELLAMY_102 [*Synechococcus* phage Bellamy] | | 7.00E-18 | 79% | |
| 7 | + | 2347 | 2538 | 63 | hypothetical protein CBD74_07960 [*Saprospirales* bacterium TMED214] | | 1.00E-18 | 58% | |
| | | | | | hypothetical protein SSM2_090 [*Synechococcus* phage S-SM2] | | 3.81E-17 | 67.3% | |
| 8 | + | 2587 | 3360 | 257 | hypothetical protein SSM2_091 [*Synechococcus* phage S-SM2] | | 1.00E-167 | 86% | |
| 10 | + | 3512 | 4678 | 388 | tryptophan halogenase [Cyanophage P-TIM40] | tryptophan halogenase (*prnA*) | 4.00E-57 | 37% | pfam04820 (3.10E-14) |

| | | | | | | | | | |
|---|---|---|---|---|---|---|---|---|---|
| 11 | + | 4705 | 4854 | 49 | hypothetical protein LIS021110_084 [Cyanophage S-RIM14] | | 5.00E-12 | 71% | PHA02324 (3.59E-07) |
| 12 | + | 5022 | 5735 | 237 | phosphoribosylaminoimidazole synthetase [*Euryarchaeota archaeon* TMED129] | | 1.00E-148 | 88% | |
| | | | | | phosphoribosylaminoimidazole synthetase [*Synechococcus* phage S-SM2] | | 1.21E-147 | 87.4% | |
| 13 | + | 5776 | 6177 | 133 | base plate wedge subunit [uncultured Mediterranean phage] | baseplate wedge subunit (T4-like gp25) | 7.00E-38 | 54% | PHA00415 (6.69E-20) |
| | | | | | putative baseplate wedge protein [*Prochlorococcus* phage P-TIM68] | | 3.43E-36 | 53.4% | |
| 14 | + | 6178 | 8124 | 648 | baseplate wedge [*Synechococcus* phage S-SM2] | baseplate wedge subunit (T4-like gp6) | 0 | 52% | PHA02553 (4.27E-105) |
| 15 | + | 8137 | 20499 | 4120 | baseplate wedge initiator [*Synechococcus* phage Bellamy] | baseplate wedge subunit (T4-like gp7) | 0 | 36% | PHA02579 (6.02e-09) |
| 16 | + | 20510 | 21061 | 183 | hypothetical protein CBD57_01235 [Candidatus *Pelagibacter* sp. TMED197] | | 4.00E-12 | 37% | |
| 17 | + | 21084 | 21863 | 259 | hypothetical protein SWZG_00244 [*Synechococcus* phage S-SKS1] | baseplate wedge protein (T4-like gp10) | 1.00E-12 | 48% | |
| | | | | | phage tail fiber-like protein [*Synechococcus* phage S-SM2] | | 3.15E-10 | 47.1% | |
| 18 | + | 21888 | 23456 | 522 | hypothetical protein CBB96_01045 [Gammaproteobacteria bacterium TMED36] | baseplate wedge protein (T4-like gp8) | 0 | 61% | pfam09215 (1.68E-08) |
| | | | | | putative baseplate wedge [*Prochlorococcus* phage P-TIM68] | | 0 | 54.1% | |
| 19 | + | 23462 | 31273 | 2603 | hypothetical protein CBB96_00010 [Gammaproteobacteria bacterium TMED36] | virulence associated protein VrlC | 0 | 43% | pfam16075 (1.94E-10) |

| | | | | | | | | | |
|---|---|---|---|---|---|---|---|---|---|
| | | | | | VrlC protein [*Synechococcus* phage S-SM2] | | 0 | 43% | |
| 20 | + | 31275 | 31493 | 72 | hypothetical protein PSSM2_116 [uncultured phage MedDCM-OCT-S04-C93] | | 1.00E-06 | 71% | |
| | | | | | hypothetical protein SWPG_00010 [*Synechococcus* phage S-CBM2] | | 2.24E-12 | 50% | |
| 21 | + | 31475 | 31861 | 128 | hypothetical protein CBD63_00380 [Candidatus *Pelagibacter* sp. TMED203] | | 1.00E-38 | 49% | |
| | | | | | hypothetical protein PBI_BELLAMY_116 [*Synechococcus* phage Bellamy] | | 1.10E-36 | 52.3% | |
| 22 | + | 31884 | 32726 | 280 | hypothetical protein CBE21_10535 [Proteobacteria bacterium TMED261] | neck protein (T4-like gp13) | 3.00E-139 | 70% | PHA02554 (2.55E-41) |
| | | | | | neck protein [*Synechococcus* phage Bellamy] | | 5.00E-139 | 68% | |
| 23 | + | 32731 | 34179 | 482 | hypothetical protein CBC15_00680 [Candidatus *Endolissoclinum* sp. TMED55] | neck protein (T4-like gp14) | 3.00E-146 | 50% | PHA02555 (1.11E-46) |
| | | | | | putative neck protein [*Prochlorococcus* phage P-TIM68] | | 4.00E-144 | 54% | |
| 24 | + | 34200 | 35213 | 337 | hypothetical protein CBB96_08755 [Gammaproteobacteria bacterium TMED36] | tail sheath stabilization (T4-like gp15) | 1.00E-128 | 57% | PHA02556 (5.45E-52) |
| | | | | | tail sheath stabilizer [*Synechococcu*s phage Bellamy] | | 6.54E-128 | 58.1% | |
| 25 | + | 35213 | 35668 | 151 | terminase small subunit [*Synechococcus* phage Bellamy] | terminase small subunit (*terS*) (T4-like gp16) | 4.00E-48 | 62% | PHA02585 (6.41E-05) |
| 26 | + | 35707 | 40143 | 1478 | hypothetical protein Syn7803US26_253 [*Synechococcus* phage ACG-2014f] | T4-like gp37 | 7.00E-146 | 38% | |

| | | | | | | | | | |
|---|---|---|---|---|---|---|---|---|---|
| | | | | | phage tail fiber-like protein [uncultured Mediterranean phage uvMED] | | 9.00E-113 | 43% | |
| 27 | + | 40147 | 40485 | 112 | hypothetical protein Syn7803C90_266 [*Synechococcus* phage ACG-2014f] | | 2.00E-15 | 42% | |
| 28 | + | 40501 | 42636 | 711 | hypothetical protein CBC18_03130 [Deltaproteobacteria bacterium TMED58] | tail fiber protein | 2.00E-59 | 39% | pfam13884 (2.44e-13) |
| | | | | | tail fiber protein [*Synechococcus* phage S-CAM9] | | 6.00E-55 | 38% | |
| 29 | + | 42662 | 44215 | 517 | hypothetical protein PBI_BELLAMY_123 [*Synechococcus* phage Bellamy] | | 2.00E-105 | 59% | |
| 30 | + | 44215 | 44535 | 106 | hypothetical protein LIS121010_102 [*Synechococcus* phage S-RIM2] | | 3.00E-37 | 66% | |
| 31 | + | 44538 | 45287 | 249 | hypothetical protein SWZG_00150 [*Synechococcus* phage S-SKS1] | | 6.00E-118 | 77% | |
| 32 | + | 45295 | 46947 | 550 | terminase large subunit [uncultured Mediterranean phage uvMED] | terminase large subunit (*terL*) (T4-like gp17) | 0 | 89% | PHA02533 (2.18E-176 |
| | | | | | terminase DNA packaging enzyme large subunit [*Synechococcus* phage S-SM2] | | 0 | 89.3% | |
| 33 | + | 46947 | 47168 | 73 | hypothetical protein [uncultured Mediterranean phage uvMED] | | 2.00E-35 | 81% | |
| | | | | | T4-like endonuclease VII [*Synechococcus* phage S-PM2] | | 3.51E-20 | 52.6% | |
| 34 | + | 47247 | 49412 | 721 | tail sheath monomer [*Synechococcus* phage S-SM2] | tail sheath monomer (T4-like gp18) | 0 | 61% | PHA02539 (0) |
| 35 | + | 49456 | 50103 | 215 | phage tail tube protein [uncultured Mediterranean phage uvMED] | tail tube protein (T4-like gp19) | 1.00E-79 | 61% | PHA02551 (7.17E-39) |

| | | | | | | | | | |
|---|---|---|---|---|---|---|---|---|---|
| | | | | | putative tail tube monomer [*Prochlorococcus* phage P-TIM68] | | 1.57E-69 | 56.2% | |
| 36 | + | 50139 | 51812 | 557 | portal protein [*Synechococcus* phage Bellamy] | portal protein (T4-like gp20) | 0 | 81% | PHA02531 (3.06E-165) |
| 37 | + | 51854 | 52009 | 51 | hypothetical protein [uncultured Mediterranean phage] | | 7.00E-13 | 71% | |
| | | | | | hypothetical protein PBI_BELLAMY_129 [*Synechococcus* phage Bellamy] | | 3.14E-10 | 49% | |
| 40 | + | 52706 | 53356 | 216 | prohead core scaffold and protease [uncultured Mediterranean phage uvMED] | prohead protease (T4-like gp21) | 2.00E-113 | 78% | PHA00911 (4.63E-48) |
| | | | | | putative prohead protease [*Prochlorococcus* phage P-TIM68] | | 2.85E-113 | 71.3% | |
| 41 | + | 53437 | 54579 | 380 | T4 prohead core scaffold protein [*Euryarchaeota archaeon* TMED129] | prohead core scaffold protein (T4-like gp22) | 2.00E-143 | 69% | PHA02557 (8.19E-52) |
| | | | | | scaffolding protein [*Synechococcus* phage Bellamy] | | 5.14E-152 | 71.2% | |
| 42 | + | 54627 | 56024 | 465 | major capsid protein [uncultured Mediterranean phage uvMED] | major capsid protein (T4-like gp23) | 0 | 83% | PHA02541 (3.05E-119) |
| | | | | | precursor of major head subunit [*Synechococcus* phage S-SSM7] | | 0 | 79.3% | |
| 45 | + | 56699 | 57289 | 196 | phage tail tube protein [uncultured Mediterranean phage uvMED] | tail tube protein (T4-like gp3) | 1.00E-73 | 60% | PHA02576 (7.08E-09) |
| | | | | | head-proximal tip of tail tube tail completion + sheath stabilizer protein [*Synechococcus* phage S-SM2] | | 1.49E-66 | 60.2% | |

| | | | | | | | | | |
|---|---|---|---|---|---|---|---|---|---|
| 46 | + | 57299 | 57736 | 145 | UsvY [*Synechococcus* phage Bellamy] | UsvY (T4-like) | 5.00E-72 | 71% | pfam11056 (1.59E-27) |
| 47 | + | 57729 | 59192 | 487 | RNA-DNA + DNA-DNA helicase [*Synechococcus* phage S-SM2] | UvsW (T4-like) | 0 | 74% | PHA02558 (3.14E-132) |
| 48 | + | 59192 | 59581 | 129 | hypothetical protein [uncultured Mediterranean phage uvMED] | | 1.00E-34 | 51% | |
| | | | | | methylamine utilization protein [*Synechococcus* phage S-SM2] | | 3.00E-32 | 44% | |
| 49 | + | 59688 | 60185 | 165 | sigma factor for late transcription [uncultured Mediterranean phage uvMED] | sigma factor for late transcription (T4-like gp55) | 4.00E-80 | 79% | PHA02547 (9.08E-29) |
| | | | | | late transcription sigma factor [*Synechococcus* phage Bellamy] | | 2.99E-82 | 71.5% | |
| 50 | + | 60182 | 61231 | 349 | recombination endonuclease subunit [*Synechococcus* phage S-SM2] | endonuclease (T4-like gp47) | 3.00E-170 | 69% | PHA02546 (7.62E-71) |
| 51 | + | 61233 | 61502 | 89 | hypothetical protein SSM2_129 [*Synechococcus* phage S-SM2] | | 6.00E-27 | 48% | pfam11360 (3.82E-10) |
| 52 | + | 61489 | 63204 | 571 | recombination endonuclease subunit [uncultured Mediterranean phage uvMED] | endonuclease (T4-like gp46) | 0 | 62% | PHA02562 (2.24E-148) |
| | | | | | recombination endonuclease [*Synechococcus* phage Bellamy] | | 0 | 61.6% | |
| 53 | + | 63201 | 64046 | 281 | protein-tyrosine sulfotransferase 2 like protein [uncultured Mediterranean phage uvMED] | sulfotransferase | 6.00E-55 | 37% | pfam13469 (3.50E-17) |
| | | | | | sulfotransferase [*Burkholderia* sp. lig30] | | 3.89E-28 | 27% | |

| | | | | | | | | | |
|---|---|---|---|---|---|---|---|---|---|
| | | | | | sulfotransferase [*Synechococcus* sp. WH 8016] | | 2.95E-18 | 24.1% | |
| 54 | + | 64060 | 64380 | 106 | hypothetical protein PSSM2_149 [*Prochlorococcus* phage P-SSM2] | | 2.00E-26 | 55% | |
| 55 | + | 64405 | 66540 | 711 | peptidase [uncultured Mediterranean phage uvMED] | | 0 | 56% | |
| | | | | | peptidase [*Synechococcus* phage S-SM2] | | 0 | 54.7% | |
| 56 | + | 66537 | 67175 | 212 | hypothetical protein CBD57_02535 [Candidatus *Pelagibacter* sp. TMED197] | class I SAM-dependent methyltransferase | 4.00E-24 | 34% | pfam13578 (4.98E-08) |
| | | | | | hypothetical protein CPRG_00165 [Cyanophage Syn30] | | 1.08E-15 | 30.6% | |
| 58 | + | 67371 | 68486 | 371 | putative cobalt chelatase subunit CobS [uncultured Mediterranean phage uvMED] | cobalt chelatase subunit (*cobS*) | 0 | 80% | PHA02244 (3.87E-18) |
| | | | | | CobS-like protein [*Synechococcus* phage Bellamy] | | 0 | 73.1% | |
| 59 | + | 68507 | 69013 | 168 | hypothetical protein CBD74_13015 [*Saprospirales* bacterium TMED214] | | 5.00E-69 | 88% | pfam11753 (9.22E-06) |
| | | | | | hypothetical protein N161109_130 [*Synechococcus* phage S-CAM9] | | 7.05E-56 | 65.6% | |
| 60 | + | 69000 | 69668 | 222 | sliding clamp DNA polymerase accessory protein [*Synechococcus* phage S-SM2] | DNA polymerase sliding clamp (T4-like gp45) | 1.00E-111 | 75% | PHA02545 (5.94E-51) |
| 61 | + | 69658 | 70263 | 201 | DNA endonuclease V [*Synechococcus* phage ACG-2014j] | | 1.00E-35 | 39% | |
| 62 | + | 70260 | 70787 | 175 | hypothetical protein [uncultured Mediterranean phage uvMED] | | 6.00E-123 | 94% | |

| | | | | | | | | | |
|---|---|---|---|---|---|---|---|---|---|
| | | | | | hypothetical protein Syn7803C88_118 [*Synechococcus* phage ACG-2014d] | | 1.14E-93 | 76.6% | |
| 63 | + | 70789 | 71295 | 168 | hypothetical protein R1080702_023 [Cyanophage S-RIM32] | 2OG-Fe (II) oxygenase superfamily protein | 7.00E-13 | 42% | pfam13661 (3.89E-12) |
| 64 | + | 71339 | 71857 | 172 | NUDIX hydrolase [*Parcubacteria* group bacterium GW2011_GWA1_40_21] | GDP-mannose glycosyl hydrolase | 5.00E-13 | 35% | cd03430 (2.33E-10) |
| | | | | | hypothetical protein CPRG_00124 [Cyanophage Syn30] | | 1.25E-08 | 73.5% | |
| 65 | + | 71860 | 72810 | 316 | DNA polymerase clamp loader small subunit [*Synechococcus* phage S-CAM9] | DNA polymerase clamp loader (T4-like gp44) | 0 | 81% | PHA02544 (9.02E-89) |
| 66 | + | 72807 | 73004 | 65 | hypothetical protein CYVG_00043 [Cyanophage S-SSM6a] | | 4.00E-17 | 61% | |
| 67 | + | 73006 | 73299 | 97 | hypothetical protein Syn7803US105_138 [*Synechococcu*s phage ACG-2014g] | | 2.00E-31 | 58% | |
| 68 | + | 73315 | 74190 | 291 | hypothetical protein SWZG_00111 [*Synechococcus* phage S-SKS1] | DNA adenine-methylase (T4-like) | 0 | 90% | COG0338 (1.52E-16) |
| 69 | + | 74181 | 74561 | 126 | clamp loader subunit [uncultured Mediterranean phage uvMED] | clamp loader small subunit | 8.00E-65 | 73% | PHA02593 (3.23E-06) |
| | | | | | clamp loader subunit [*Synechococcus* phage S-SM2] | | 5.34E-59 | 68.3% | |
| 70 | + | 74558 | 74992 | 144 | endoribonuclease translational repressor of early genes [*Synechococcus* phage S-CAM9] | translation repressor (T4-like) | 2.00E-78 | 78% | PHA02543 (4.73E-60) |
| 71 | + | 74989 | 75564 | 191 | hypothetical protein PSSM7_086 [*Prochlorococcus* phage P-SSM7] | 2OG-Fe (II) oxygenase superfamily protein | 2.00E-53 | 48% | pfam13759 (3.71E-05) |
| 72 | + | 75668 | 76102 | 144 | heat shock protein [uncultured Mediterranean phage uvMED] | heat shock protein (*hsp20*) | 9.00E-97 | 94% | PRK10743 (6.18E-26) |

| | | | | | | | | | |
|---|---|---|---|---|---|---|---|---|---|
| | | | | | heat shock protein [*Synechococcus* phage S-SM2] | | 1.24E-79 | 75.7% | |
| 73 | + | 76202 | 76576 | 124 | hypothetical protein SSM2_148 [*Synechococcus* phage S-SM2] | | 1.00E-23 | 42% | |
| 74 | + | 76563 | 76781 | 72 | hypothetical protein PBI_BELLAMY_160 [*Synechococcus* phage Bellamy] | | 3.00E-27 | 66% | |
| 75 | + | 76778 | 79273 | 831 | DNA polymerase [*Synechococcus* phage S-SM2] | DNA polymerase (T4-like gp43) | 0 | 82% | PHA02528 (0) |
| 76 | + | 79270 | 79467 | 65 | hypothetical protein N161109_142 [*Synechococcus* phage S-CAM9] | | 1.00E-37 | 94% | |
| 77 | + | 79464 | 80204 | 246 | hypothetical protein [uncultured Mediterranean phage uvMED] | 2OG-Fe (II) oxygenase superfamily protein | 1.00E-23 | 33% | pfam13759 (5.93E-07) |
| | | | | | hypothetical protein CPXG_00181 [Cyanophage P-RSM6] | | 3.21E-05 | 32.5% | |
| 78 | + | 80211 | 81227 | 338 | UvsX RecA-like protein [uncultured Mediterranean phage uvMED] | recombinase A (*uvsX*) (T4-like) | 0 | 88% | PRK09354 (2.17E-18) |
| | | | | | recombination protein [*Synechococcus* phage S-CAM9] | | 0 | 84.9% | |
| 79 | + | 81292 | 81876 | 194 | hypothetical protein [*Prochlorococcus* phage P-TIM68] | 2OG-Fe (II) oxygenase superfamily protein | 3.00E-52 | 44% | pfam13759 (1.26E-15) |
| 80 | + | 81879 | 82595 | 238 | hypothetical protein [*Prochlorococcus* phage P-TIM68] | 2OG-Fe (II) oxygenase superfamily protein | 4.00E-52 | 42% | pfam13759 (4.14E-12) |
| 81 | + | 82549 | 83925 | 458 | DNA primase [*Hyphomonas* sp. TMED31] | DNA primase (T4-like gp41) | 0 | 84% | PHA02542 (8.02E-163) |
| | | | | | DNA helicase/primase [*Synechococcus* phage Bellamy] | | 0 | 82% | |

| | | | | | | | | | |
|---|---|---|---|---|---|---|---|---|---|
| 82 | + | 83925 | 84173 | 82 | hypothetical protein PBI_BELLAMY_166 [*Synechococcus* phage Bellamy] | | 1.00E-19 | 65% | |
| 83 | + | 84170 | 84634 | 154 | pyrophosphatase [Cyanophage S-RIM50] | pyrophosphohydrolase (*mazG*) | 9.00E-74 | 80% | cd11541 (5.47E-16) |
| 84 | + | 84631 | 85167 | 178 | hypothetical protein Syn7803US79_154 [*Synechococcus* phage ACG-2014a] | | 2.00E-32 | 40% | |
| | | | | | DNA endonuclease V [*Synechococcus* phage S-MbCM100] | | 2.00E-32 | 40% | |
| 85 | + | 85160 | 85303 | 47 | gp29 [*Synechococcus* phage syn9] | | 3.00E-17 | 78% | |
| 86 | + | 85300 | 85524 | 74 | hypothetical protein S-MbCM7_214 [*Synechococcus* phage ACG-2014h] | | 5.00E-28 | 72% | |
| 87 | + | 85508 | 85747 | 79 | hypothetical protein [Cyanophage S-TIM5] | | 2.00E-23 | 78% | |
| 88 | + | 85744 | 86328 | 194 | 2OG-Fe(II) oxygenase [*Synechococcus* phage S-H35] | 2OG-Fe (II) oxygenase superfamily protein | 2.00E-61 | 55% | pfam13640 (6.19E-08) |
| 89 | + | 86357 | 87328 | 323 | hypothetical protein SWYG_00152 [*Synechococcus* phage S-IOM18] | | 7.00E-43 | 83% | pfam04006 (1.21E-05) |
| 90 | + | 87458 | 88792 | 444 | hypothetical protein SWPG_00070 [*Synechococcus* phage S-CBM2] | | 2.00E-120 | 52% | |
| 91 | + | 88819 | 90048 | 409 | cytitidyltransferase [*Synechococcus* phage S-SM2] | Nicotinamide/nicotinate mononucleotide adenylyltransferase (NMNAT) | 0 | 70% | cd02167 (5.81E-05) |
| 92 | + | 90061 | 91623 | 520 | hypothetical protein PBI_BELLAMY_171 [*Synechococcus* phage Bellamy] | | 1.00E-20 | 33% | |

| | | | | | | | | | |
|---|---|---|---|---|---|---|---|---|---|
| 93 | + | 91626 | 91835 | 69 | hypothetical protein SSSM7_236 [*Synechococcus* phage S-SSM7] | | 9.00E-06 | 63% | |
| 94 | + | 91852 | 92064 | 70 | hypothetical protein C421010_183 [*Synechococcus* phage S-CAM3] | | 8.00E-35 | 89% | PHA02334 (8.97E-34) |
| 95 | + | 92158 | 92691 | 177 | hypothetical protein [uncultured Mediterranean phage uvMED] | | 1.00E-39 | 45% | |
| | | | | | hypothetical protein SSM2_165 [*Synechococcus* phage S-SM2] | | 6.65E-39 | 54.7% | |
| 96 | + | 92703 | 93095 | 130 | hypothetical protein SSM2_166 [*Synechococcus* phage S-SM2] | | 6.73E-61 | 70.6% | PHA02335 (8.05E-42) |
| 97 | + | 93121 | 93306 | 61 | hypothetical protein SShM2_188 [*Synechococcus* phage S-ShM2] | | 6.00E-05 | 61% | |
| 98 | + | 93281 | 93712 | 143 | hypothetical protein SXBG_00144 [*Synechococcus* phage S-CAM1] | | 9.00E-51 | 73% | |
| 99 | + | 93699 | 94667 | 322 | DNA primase [Candidatus *Endolissoclinum* sp. TMED55] | DNA primase (T4-like gp61) | 4.00E-156 | 63% | PHA02540 (6.26E-83) |
| | | | | | DNA primase subunit [*Synechococcus* phage S-SKS1] | | 4.00E-155 | 66% | |
| 100 | + | 94664 | 96961 | 765 | ribonucleotide-diphosphate reductase subunit alpha [Candidatus *Thorarchaeota archaeon* SMTZ-45] | ribonucleotide-diphosphate reductase subunit alpha (*nrdA*) (T4-like) | 0 | 85% | PHA02572 (0) |
| | | | | | ribonucleotide reductase class Ia alpha subunit [*Synechococcus* phage S-SM2] | | 0 | 84.1% | |
| 101 | + | 96942 | 98132 | 396 | NrdB-like protein [*Synechococcus* phage Bellamy] | ribonucleotide-diphosphate reductase subunit beta (*nrdB*) (T4-like) | 0 | 85.9% | PRK09101 (9.72E-135) |

| | | | | | | | | | |
|---|---|---|---|---|---|---|---|---|---|
| 103 | + | 98328 | 98756 | 142 | NAD synthetase [*Synechococcus* phage ACG-2014d] | GIY-YIG endonucleases (T4-like) | 5.00E-73 | 77% | cd10444 (6.39E-06) |
| 106 | + | 99752 | 99916 | 54 | hypothetical protein T191209_025 [*Synechococcus* phage S-CAM22] | | 3.00E-05 | 63% | |
| 107 | + | 100204 | 100881 | 225 | hypothetical protein CBD83_08460 [*Prochlorococcus* sp. TMED223] | 2OG-Fe(II) oxygenase superfamily protein | 2.00E-40 | 37% | pfam13759 (4.48e-11) |
| 108 | + | 101127 | 101294 | 55 | hypothetical protein S330809_181 [*Synechococcus* phage S-CAM4] | | 2.00E-16 | 51% | |
| 109 | + | 101502 | 101729 | 75 | hypothetical protein [*Synechococcus* phage S-PM2] | | 7.00E-26 | 73% | PHA02325 (2.10E-35) |
| 110 | + | 101729 | 101893 | 54 | hypothetical protein S-MbCM7_114 [*Synechococcus* phage ACG-2014h] | | 7.00E-18 | 57% | |
| 111 | + | 101893 | 102069 | 58 | hypothetical protein SWTG_00079 [*Synechococcus* phage S-RIM2 R1_1999] | | 5.00E-22 | 83% | |
| 112 | + | 102203 | 102787 | 194 | 2OG-Fe(II) oxygenase [*Synechococcus* phage S-CAM8] | 2OG-Fe (II) oxygenase superfamily protein | 2.00E-37 | 38% | pfam13640 (1.96E-23) |
| 113 | + | 102789 | 103928 | 379 | predicted protein [*Thalassiosira pseudonana* CCMP1335] | GDP-fucose protein O-fucosyltransferase | 1.00E-07 | 27% | cd11296 (2.97E-07) |
| 114 | + | 103925 | 104620 | 231 | conserved hypothetical protein [*Synechococcus* sp. WH 8102] | | 1E-56 | 44% | |
| 115 | + | 104838 | 105053 | 71 | hypothetical protein CBC89_01995 [*Euryarchaeota archaeon* TMED129] | | 4.00E-35 | 80% | PHA02360 (3.97E-27) |
| | | | | | hypothetical protein PBI_BELLAMY_197 [*Synechococcus* phage Bellamy] | | 1.55E-33 | 82.6% | |
| 116 | + | 105053 | 105286 | 77 | hypothetical protein PBI_BELLAMY_198 [*Synechococcus* phage Bellamy] | | 1.00E-45 | 91% | |

| | | | | | | | | | |
|---|---|---|---|---|---|---|---|---|---|
| 117 | + | 105322 | 106872 | 516 | hypothetical protein [*Jiulongibacter sediminis*] | carbamoyl transferase (*nol*) | 7.00E-124 | 41% | COG2192 (6.97E-59) |
| | | | | | carbamoyl transferase [*Xanthomonas* phage XacN1] | | 6.00E-100 | 40% | |
| 118 | + | 106869 | 107216 | 115 | hypothetical protein CBC18_00390 [Deltaproteobacteria bacterium TMED58] | mannose-6-phosphate isomerase (MPI) | 8.00E-28 | 45% | COG0662 (2.94E-22) |
| 120 | + | 107672 | 108286 | 204 | hypothetical protein SXBG_00053 [*Synechococcus* phage S-CAM1] | 2OG-Fe (II) oxygenase superfamily protein | 7.00E-36 | 40% | pfam13759 (1.65E-09) |
| 121 | + | 108279 | 109307 | 342 | GDP-mannose 4,6-dehydratase [*Planctomycetia* bacterium TMED53] | GDP-mannose 4,6-dehydratase | 0 | 77% | COG1089 (3.07E-174) |
| | | | | | GDP-D-mannose dehydratase [*Prochlorococcus* phage P-SSM2] | | 0 | 76.4% | |
| 122 | + | 109297 | 110301 | 334 | GDP-L-fucose synthase [*Thermus aquaticus*] | GDP-L-fucose synthase | 4.00E-137 | 61% | PLN02725 (2.55E-133) |
| 123 | + | 110305 | 111294 | 329 | putative NAD-dependent epimerase [*Prochlorococcus* phage P-TIM68] | GDP-mannose-3',5'-epi meras | 3.00E-158 | 66% | cd05273 (6.53E-103) |
| 124 | + | 111285 | 112136 | 283 | hypothetical protein [*Massilia* sp. Root351] | | 1.00E-38 | 36% | |
| 125 | + | 112133 | 113002 | 289 | hypothetical protein CK425_10975 [*Parachlamydia* sp.] | | 8.00E-53 | 38% | |
| | | | | | hypothetical protein PBI_BELLAMY_220 [*Synechococcus* phage Bellamy] | | 3.04E-33 | 30.7% | |
| 126 | + | 112999 | 113787 | 262 | alpha-1,2-fucosyltransferase [Rhodospirillaceae bacterium TMED167] | alpha-1,2-fucosyltransfer ase | 7.00E-114 | 59% | cd11301 (1.62E-28) |
| | | | | | glycosyltransferase family 11 [*Synechococcus* phage S-SM2] | | 1.20E-105 | 57.6% | |

| | | | | | | | | | |
|---|---|---|---|---|---|---|---|---|---|
| 127 | + | 113784 | 114536 | 250 | hypothetical protein CBC83_08770 [Flavobacteriales bacterium TMED123] | glycosyltransferase | 2.00E-56 | 45% | cd04184 (1.84E-12) |
| 128 | + | 114523 | 115362 | 279 | hypothetical protein S420910_232 [*Synechococcus* phage S-CAM7] | glycosyltransferase | 3.00E-106 | 57% | COG1215 (7.55e-05) |
| 129 | + | 115338 | 116030 | 230 | hypothetical protein CBC83_08780 [Flavobacteriales bacterium TMED123] | | 1.00E-105 | 67% | pfam04765 (3.18E-16) |
| 130 | + | 116030 | 117352 | 440 | hypothetical protein CBD58_01790 [bacterium TMED198] | asparagine synthase | 5.00E-150 | 50% | COG0367 (2.09E-21) |
| | | | | | asparagine synthase [*Clostridium josui*] | | 8.00E-126 | 47% | |
| 131 | + | 117352 | 117897 | 181 | hypothetical protein [*Moorea producens*] | methyltransferase | 3.00E-39 | 44% | COG2521 (6.64E-05) |
| 132 | + | 117900 | 118700 | 266 | hypothetical protein [*Paenibacillus* sp. BIHB4019] | | 2.00E-16 | 29% | |
| 133 | + | 118687 | 119274 | 195 | CAZy families GT2\|GT4 protein [uncultured *Burkholderia* sp.] | methyltransferase | 2.00E-18 | 31% | pfam13578 (1.12e-06) |
| | | | | | class I SAM-dependent methyltransferase [*alpha proteobacterium* L41A] | | 3.20E-16 | 32.3% | |
| 134 | + | 119271 | 120062 | 263 | hypothetical protein [*Polyangium*] *brachysporum*] | | 2.00E-31 | 30% | |
| 135 | + | 120055 | 120840 | 261 | putative glycosyltransferase [*Prochlorococcus* phage P-TIM68] | glycosyltransferase | 4.00E-143 | 69% | cd06532 (1.17E-11) |
| 136 | + | 120898 | 121116 | 72 | high light inducible protein [*Synechococcus* phage S-SM1] | high light inducible protein (*hli*) | 6.00E-34 | 88% | PHA02337 (5.36E-06) |
| 137 | + | 121208 | 121501 | 97 | ferredoxin [Cyanophage S-RIM50] | ferredoxin | 9.00E-44 | 89% | TIGR02008 (4.85E-42) |

| | | | | | | | | | |
|---|---|---|---|---|---|---|---|---|---|
| 138 | + | 121501 | 121662 | 53 | hypothetical protein C421010_217 [*Synechococcus* phage S-CAM3] | | 2.00E-18 | 74% | |
| 139 | + | 121659 | 121766 | 35 | high light inducible protein [*Synechococcus* phage ACG-2014d] | high light inducible protein (*hli*) | 2.00E-14 | 91% | PHA02337 (1.24E-13) |
| 140 | + | 121809 | 122447 | 212 | hypothetical protein CYXG_00180 [*Synechococcus* phage S-SSM4] | peroxiredoxin | 1.00E-60 | 54% | COG0678 (3.35E-52) |
| 141 | + | 122561 | 123166 | 201 | hypothetical cyanophage protein [*Synechococcus* phage S-RSM4] | | 1.00E-115 | 80% | |
| 142 | + | 123163 | 123540 | 125 | hypothetical protein SSM2_228 [*Synechococcus* phage S-SM2] | | 6.00E-37 | 59% | |
| 143 | + | 123687 | 124058 | 123 | DUF680 domain-containing protein [*Synechococcus* phage S-ShM2] | | 4.00E-44 | 75% | |
| 144 | + | 124256 | 124615 | 119 | hypothetical protein CBC89_02510 [*Euryarchaeota archaeon* TMED129] | | 1.00E-41 | 71% | |
| | | | | | DUF680 domain-containing protein [*Synechococcus* phage S-SSM5] | | 5.96E-45 | 63.6% | |
| 145 | + | 124625 | 125599 | 324 | phosphate ABC transporter substrate-binding protein [Cyanophage S-TIM5] | phosphate ABC transporter substrate-binding protein (*pstS*) | 0 | 88% | TIGR00975 (9.75E-76) |
| | | | | | ABC-type phosphate transport system [*Synechococcus* phage S-SKS1] | | 0 | 78.9% | |
| 146 | + | 125610 | 125876 | 88 | hypothetical protein SSM2_222 [*Synechococcus* phage S-SM2] | | 7.00E-19 | 56% | |
| 147 | + | 125896 | 126180 | 94 | hypothetical protein [uncultured Mediterranean phage uvMED] | | 9.00E-50 | 78% | |

| | | | | | | | | |
|---|---|---|---|---|---|---|---|---|
| | | | | | hypothetical protein Syn7803US103_222 [*Synechococcus* phage ACG-2014j] | | 1.91E-12 | 50% | |
| 148 | + | 126146 | 126982 | 278 | hypothetical protein Syn7803C100_17 [*Synechococcus* phage ACG-2014b] | | 6.00E-113 | 76% | |
| 149 | + | 127159 | 127443 | 94 | hypothetical protein P29A0810_138 [*Synechococcus* phage S-CAM8] | | 3.00E-35 | 81% | |
| 150 | + | 127444 | 127659 | 71 | hypothetical protein CBC89_04105 [*Euryarchaeota archaeon* TMED129] | | 4.00E-18 | 59% | cd10973 (7.43E-05) |
| | | | | | hypothetical protein C421010_121 [*Synechococcus* phage S-CAM3] | | 4.66E-09 | 75% | |
| 151 | + | 127683 | 128006 | 107 | plastocyanin [*Synechococcus* phage S-RIM2 R1_1999] | plastocyanin (*petE*) | 9.81E-67 | 91% | PRK02710 (5.22E-32) |
| 153 | + | 128127 | 128630 | 167 | alternative oxidase-like protein [uncultured Mediterranean phage uvMED] | plastoquinol terminal oxidase (PTOX) | 9.74E-105 | 87% | cd01053 (7.12E-06) |
| | | | | | plastoquinol terminal oxidase [*Synechococcus* phage Bellamy] | | 6.32E-103 | 85% | |
| 154 | + | 128715 | 128858 | 47 | hypothetical protein SSM2_238 [*Synechococcus* phage S-SM2] | | 1.00E-13 | 74% | |
| 155 | + | 128855 | 129907 | 350 | 6-phosphogluconate dehydrogenase [*Synechococcus* phage S-RIM2] | 6-phosphogluconate dehydrogenase (*gnd*) | 0 | 84% | PRK09599 (1.5E-90) |
| 156 | + | 130003 | 131340 | 445 | glucose-6-phosphate dehydrogenase [uncultured Mediterranean phage uvMED] | glucose-6-phosphate dehydrogenase (*zwf*) | 0 | 92% | PRK05722 (2.74E-120) |
| | | | | | glucose-6-phosphate dehydrogenase [*Synechococcus* phage S-SM2] | | 0 | 80.3% | |
| 157 | + | 131337 | 131498 | 53 | hypothetical protein SSM2_242 [*Synechococcus* phage S-SM2] | | 1.00E-20 | 75% | |

| | | | | | | | | | |
|---|---|---|---|---|---|---|---|---|---|
| 158 | + | 131495 | 131728 | 77 | hypothetical protein SWYG_00084 [*Synechococcus* phage S-IOM18] | | 2.00E-34 | 81% | |
| | | | | | hypothetical protein SWZG_00060 [*Synechococcus* phage S-SKS1] | | 2.58E-28 | 83.1% | |
| 159 | + | 131704 | 131913 | 69 | hypothetical protein PBI_BELLAMY_137 [*Synechococcus* phage Bellamy] | | 7.00E-31 | 84% | |
| 160 | + | 131918 | 132127 | 69 | hypothetical protein [uncultured Mediterranean phage uvMED] | | 1.00E-34 | 87% | |
| | | | | | hypothetical protein S820908_097 [*Synechococcus* phage S-CAM9] | | 2.44E-37 | 85.5% | |
| 161 | + | 132384 | 132845 | 153 | hypothetical protein Syn19_113 [*Synechococcus* phage Syn19] | endonuclease | 6.00E-100 | 94% | pfam00565 (5.82E-10) |
| 162 | + | 132842 | 133618 | 258 | hypothetical protein [uncultured Mediterranean phage uvMED] | | 1.00E-40 | 42% | |
| 163 | + | 133619 | 133852 | 77 | gp162 [*Synechococcus* phage syn9] | plasmid stability protein | 4.00E-31 | 63% | PHA02357 (7.9E-26) |
| 164 | + | 133856 | 134482 | 208 | hypothetical protein SXCG_00149 [*Synechococcus* phage S-CAM8] | | 3.39E-106 | 72% | PHA02358 (1.95E-41) |
| 165 | + | 134644 | 135477 | 277 | hypothetical protein Syn7803C2_181 [*Synechococcus* phage ACG-2014e] | | 1.00E-73 | 66% | |
| 166 | - | 136117 | 136554 | 145 | hypothetical protein S-MbCM7_186 [*Synechococcus* phage ACG-2014h] | | 2.00E-55 | 83% | |
| 167 | - | 136554 | 137057 | 167 | gp167 [*Synechococcus* phage syn9] | | 8.00E-57 | 81% | |
| | | | | | OMP1 protein [*Synechococcus* phage ACG-2014j] | | 6.00E-35 | 63% | |

| | | | | | | | | | |
|---|---|---|---|---|---|---|---|---|---|
| 168 | + | 137300 | 138382 | 360 | D1 protein [Cyanophage S-TIM5] | photosystem Ⅱ D1 (*psbA*) | 0 | 98% | TIGR01151 (0) |
| | | | | | photosystem II protein D1 [Cyanophage S-RIM32] | | 0 | 97% | |
| 169 | + | 138542 | 139600 | 352 | photosystem II protein D2 [*Synechococcus* phage S-WAM1] | photosystem Ⅱ D2 (*psbD*) | 0 | 96.3% | TIGR01152 (0) |
| 170 | + | 139635 | 140225 | 196 | hypothetical protein [uncultured Mediterranean phage uvMED] | | 3.00E-22 | 38% | |
| | | | | | hypothetical cyanophage protein [*Synechococcus* phage S-RSM4] | | 2.11E-10 | 32.9% | |
| 171 | + | 140222 | 140419 | 65 | gp181 [uncultured Mediterranean phage uvMED] | | 4.00E-20 | 55% | |
| | | | | | hypothetical protein R1080702_166 [Cyanophage S-RIM32] | | 5.61E-18 | 55.4% | |
| 172 | + | 140416 | 141063 | 215 | *talC*-like protein [*Synechococcus* phage Bellamy] | transaldolase-like fructose-6-phosphate aldolases (*talC*) | 2.00E-113 | 80% | PRK13247 (1.02E-54) |
| 173 | + | 141060 | 141284 | 74 | hypothetical protein SSM2_255 [*Synechococcus* phage S-SM2] | | 5.00E-25 | 71% | |
| 174 | + | 141281 | 141904 | 207 | phycoerythrobilin synthase [Cyanophage S-SSM6a] | phycoerythrobilin synthase (*pebS*) | 6.43E-92 | 65.1% | PRK13247 (2.92E-15) |
| 175 | + | 142088 | 142402 | 104 | putative endonuclease [*Synechococcu*s phage S-CAM7] | endonuclease | 2.00E-42 | 52% | cd00085 (8.74E-10) |
| 176 | + | 142507 | 142755 | 82 | glutaredoxin [*Euryarchaeota archaeon* TMED129] | glutaredoxin | 3.00E-28 | 59% | cd03418 (7.98E-09) |

| | | | | | | | |
|---|---|---|---|---|---|---|---|
| 177 | + | 142755 | 143117 | 120 | hypothetical protein PBI_BELLAMY_264 [*Synechococcus* phage Bellamy] | 5.00E-22 | 46% |
| 179 | + | 143271 | 143495 | 74 | hypothetical protein SSM2_261 [*Synechococcus* phage S-SM2] | 1.00E-09 | 54% |
| 180 | + | 143530 | 144027 | 165 | gp225 [uncultured Mediterranean phage uvMED] | 2.00E-66 | 62% |
| | | | | | hypothetical protein PSSM2_317 [*Prochlorococcus* phage P-SSM2] | 1.67E-63 | 61.1% |
| 181 | + | 144024 | 144290 | 88 | gp226 [uncultured Mediterranean phage uvMED] | 9.00E-21 | 65% |
| | | | | | hypothetical protein [*Synechococcus* phage S-PM2] | 1.07E-26 | 58.1% |
| 182 | + | 144283 | 144504 | 73 | hypothetical protein [uncultured Mediterranean phage uvMED] | 5.00E-06 | 43% |
| 183 | + | 144555 | 144764 | 69 | hypothetical protein ES420910_016 [Cyanophage S-RIM44] | 6.67E-32 | 84.4% |
| | | | | | hypothetical protein CBB91_08600 [*Hyphomonas* sp. TMED31] | 1.30E-18 | 65.1% |
| 184 | + | 144766 | 145080 | 104 | hypothetical cyanophage protein [*Synechococcus* phage S-RSM4] | 7.00E-66 | 92% |
| 186 | + | 145303 | 145917 | 204 | phytanoyl-CoA dioxygenase [*Prochlorococcus* *marinus* str. MIT 1313] | 2.00E-37 | 41% |
| 187 | + | 145892 | 146131 | 79 | glutaredoxin [*Synechococcus* phage S-WAM2] | 2.00E-46 | 90% |
| 188 | + | 146254 | 146448 | 64 | hypothetical protein SXDG_00158 [*Synechococcus* phage S-RIM8 A.HR1] | 1.00E-18 | 61% |

| | | | | | | | | | |
|---|---|---|---|---|---|---|---|---|---|
| 189 | + | 146520 | 146684 | 54 | hypothetical protein SWYG_00165 [*Synechococcus* phage S-IOM18] | | 3.00E-26 | 83% | |
| 190 | + | 146763 | 146921 | 52 | hypothetical protein SSM2_272 [*Synechococcus* phage S-SM2] | | 4.00E-08 | 72% | |
| 191 | + | 146931 | 147110 | 59 | hypothetical protein R290704_229 [Cyanophage S-RIM50] | | 3.00E-30 | 85% | |
| 192 | + | 147104 | 147517 | 137 | hypothetical protein SWZG_00108 [*Synechococcus* phage S-SKS1] | | 9.00E-28 | 48% | |
| 193 | + | 147514 | 148161 | 215 | thymidylate synthase [*Synechococcus* phage S-CAM9] | thymidylate synthase (*td*) | 1.00E-128 | 80% | PRK00847 (7.86E-31) |
| 194 | + | 148182 | 148751 | 189 | putative carbamoyltransferase [Cyanophage S-RIM44] | carbamoyltransferase (*nol*) | 4.00E-66 | 58% | pfam16861 (6.46E-42) |
| 195 | + | 148755 | 149981 | 408 | predicted carbamoyl transferase [uncultured Mediterranean phage uvMED] | carbamoyltransferase (*nol*) | 1.00E-52 | 36% | COG2192 (1.75E-05) |
| 196 | + | 149978 | 150535 | 185 | predicted carbamoyl transferase, NodU family (COG2192) [uncultured Mediterranean phage uvMED] | carbamoyltransferase (*nol*) | 3.00E-76 | 61% | pfam16861 (4.47E-47) |
| | | | | | putative carbamoyltransferase [Cyanophage S-RIM44] | | 3.42E-50 | 36.8% | |
| 197 | + | 150532 | 151764 | 410 | carbamoyltransferase [uncultured Mediterranean phage uvMED] | carbamoyltransferase (*nol*) | 6.00E-55 | 33% | pfam02543 (6.56E-09) |
| | | | | | carbamoyltransferase [*Synechococcus* phage S-CAM8] | | 8.04E-70 | 61.9% | |
| 198 | + | 151785 | 152801 | 338 | nucleotide-sugar epimerase [*Synechococcus* phage ACG-2014f] | dTDP-D-glucose 4,6-dehydratase | 0 | 76% | cd05246 (3.12E-63) |

| | | | | | | | | | |
|---|---|---|---|---|---|---|---|---|---|
| 199 | + | 152825 | 153076 | 83 | hypothetical protein SSM2_274 [*Synechococcus* phage S-SM2] | | 6.00E-33 | 71% | |
| 200 | + | 153078 | 153839 | 253 | P-starvation inducible protein [uncultured Mediterranean phage uvMED] | P-starvation inducible protein (*phoH*) | 8.00E-138 | 71% | pfam02562 (1.04E-26) |
| | | | | | P-starvation inducible protein [*Prochlorococcus* phage P-SSM2] | | 1.51E-134 | 70.7% | |
| 201 | + | 153836 | 154543 | 235 | hypothetical protein SSM2_276 [*Synechococcus* phage S-SM2] | | 5.00E-29 | 33% | |
| 202 | + | 154536 | 155207 | 223 | hypothetical protein SWZG_00272 [*Synechococcus* phage S-SKS1] | CRISPR/Cas system-associated protein Cas4, RecB-like nuclease | 2.00E-106 | 71% | cd09637 (9.06e-07) |
| | | | | | exonuclease [*Prochlorococcus* phage P-SSM2] | | 1.21E-109 | 68.6% | |
| 203 | + | 155281 | 155550 | 89 | late promoter transcription accessory protein [*Synechococcus* phage S-SM2] | late-transcription coactivator (T4-like gp33) | 2.00E-26 | 62% | pfam16805 (3.70E-15) |
| 204 | + | 155637 | 156224 | 195 | hypothetical protein CBC89_04010 [*Euryarchaeota archaeon* TMED129] | DNA helicase loader (T4-like gp59) | 2.00E-102 | 71% | PHA02559 (4.65E-30) |
| | | | | | loader of gp41 DNA helicase [*Synechococcus* phage S-SM2] | | 3.00E-91 | 62% | |
| 205 | + | 156221 | 156559 | 112 | hypothetical protein PBI_BELLAMY_3 [*Synechococcus* phage Bellamy] | | 9.00E-36 | 56% | pfam08855 (1.12E-09) |
| 206 | + | 156630 | 157553 | 307 | ssDNA binding protein [*Synechococcus* phage S-SM2] | ssDNA binding protein (T4-like gp32) | 3.00E-153 | 75% | pfam08804 (3.11e-50) |

| | | | | | | | | | |
|---|---|---|---|---|---|---|---|---|---|
| 207 | - | 157617 | 159812 | 731 | baseplatewedge component [*Synechococcus* phage S-SM2] | baseplate wedge subunit | 3.00E-65 | 48% | PHA02578 (9.81E-08) |
| 208 | - | 159832 | 160179 | 115 | hypothetical protein [*Prochlorococcus* phage P-TIM68] | | 3E-32 | 50% | |
| 209 | - | 160176 | 160625 | 149 | head completion protein [*Saprospirales* bacterium TMED214] | head completion protein (T4-like gp4) | 3E-76 | 75% | PHA02552 (9.36E-43) |
| | | | | | head completion protein [*Synechococcus* phage S-SM2] | | 1.32E-69 | 69.6% | |
| 210 | + | 160668 | 161522 | 284 | hypothetical protein SSM2_009 [*Synechococcus* phage S-SM2] | | 1.00E-48 | 41% | |
| | | | | | tail tube monomer [uncultured Mediterranean phage uvMED] | | 3.00E-13 | 30% | |
| 211 | + | 161566 | 162282 | 238 | baseplate hub subunit [S*ynechococcus* phage S-SM2] | baseplate hub subunit (T4-like gp26) | 1.00E-120 | 69% | pfam12322 (2.77e-10) |
| 212 | + | 162305 | 162469 | 54 | putative base plate hub assembly catalyst [*Prochlorococcus* phage P-TIM68] | base plate hub assembly catalyst | 5.36E-25 | 77.2% | PHA02078 (5.61E-14) |
| 213 | + | 162462 | 165314 | 950 | putative bacteriophage-related transmembrane protein [*Synechococcus* phage S-SM2] | mannosyl-glycoprotein endo-beta-N-acetylglucosaminidase | 2.00E-19 | 36% | pfam01832 (6.32e-10) |
| 214 | + | 165317 | 168712 | 1131 | hypothetical protein Syn7803US56_9 [*Synechococcus* phage ACG-2014b] | D-alanyl-D-alanine carboxypeptidase | 4.00E-18 | 55% | pfam02557 (1.72E-11) |
| | | | | | transglycosylase domain-containing protein [*Synechococcus* phage S-SM2] | | 4.00E-09 | 33% | |
| 215 | + | 168714 | 170018 | 434 | hypothetical protein [uncultured Mediterranean phage uvMED] | | 6.00E-39 | 29% | |

| | | | | | | | | | |
|---|---|---|---|---|---|---|---|---|---|
| | | | | | hypothetical protein [*Prochlorococcus* phage P-TIM68] | | 3.18E-44 | 29% | |
| 216 | + | 170023 | 170250 | 75 | carbon metabolic regulator [*Synechococcus* phage S-CAM9] | carbon metabolic regulator (CP12) | 1.00E-33 | 93% | smart01093 (5.60E-12) |
| 217 | + | 170258 | 173017 | 919 | T4-like baseplate hub and tail lysozyme [uncultured Mediterranean phage uvMED] | | 2.00E-47 | 32% | |
| | | | | | putative baseplate hub and tail lysozyme [*Prochlorococcus* phage P-TIM68] | | 6.13E-72 | 27% | |
| 218 | + | 173044 | 173628 | 194 | hypothetical protein [uncultured *Mediterranean* phage uvMED] | | 9.00E-18 | 33% | |
| | | | | | hypothetical protein SSM2_018 [*Synechococcus* phage S-SM2] | | 5.45E-17 | 32.6% | |
| 219 | + | 173631 | 174407 | 258 | hypothetical protein CBD74_09765 [*Saprospirales* bacterium TMED214] | | 2.00E-107 | 56% | |
| | | | | | hypothetical protein PBI_BELLAMY_19 [*Synechococcus* phage Bellamy] | | 1.98E-106 | 60.2% | |
| 220 | + | 174419 | 175066 | 215 | hypothetical protein N161109_017 [*Synechococcus* phage S-CAM9] | | 5.00E-58 | 65% | |
| 221 | + | 175115 | 175303 | 62 | hypothetical protein PBI_BELLAMY_21 [*Synechococcus* phage Bellamy] | | 5.00E-12 | 46% | |
| 222 | + | 175300 | 175758 | 152 | CpeT/CpcT family (DUF1001) [uncultured Mediterranean phage uvMED] | T-type phycobiliprotein lyase (*cpeT/cpcT*) | 2.00E-30 | 44% | cd16338 (5.40e-08) |
| | | | | | CpeT antenna protein [*Synechococcus* phage ACG-2014c] | | 4.38E-18 | 38.9% | |
| 223 | + | 175821 | 175985 | 54 | hypothetical protein [uncultured Mediterranean phage uvMED] | | 3.00E-19 | 82% | |

| | | | | | | | | | |
|---|---|---|---|---|---|---|---|---|---|
| | | | | | gp97 [*Synechococcus* phage S-RIM8 A.HR5] | | 4.00E-18 | 88% | |
| 224 | + | 175972 | 176529 | 185 | 2OG-Fe(II) oxygenase [*Synechococcus* phage S-CAM22] | 2OG-Fe (II) oxygenase superfamily protein | 1.13E-36 | 39% | pfam13640 (1.10E-09) |
| 225 | + | 176745 | 182306 | 1853 | fiber [*Synechococcus* phage S-SM2] | | 0 | 50% | |
| 226 | + | 182327 | 186364 | 1345 | hypothetical protein [*Methyloceanibacter stevinii*] | | 4.00E-23 | 33% | NF033849 (3.29e-05) |
| 227 | + | 186412 | 189576 | 1054 | phage tail fiber-like protein [uncultured Mediterranean phage uvMED] | | 3.00E-19 | 35% | |
| | | | | | phage tail fiber-like protein [*Prochlorococcus* phage P-SSM2] | | 1.60E-10 | 33.8% | |
| 228 | + | 189576 | 193811 | 1411 | hypothetical protein PSSM2_297 [*Prochlorococcus* phage P-SSM2] | baseplate wedge subunit | 0 | 52% | pfam13385 (4.18E-10) |
| 229 | + | 193829 | 195502 | 557 | hypothetical protein CBC18_00570 [Deltaproteobacteria bacterium TMED58] | | 3.00E-16 | 43% | |
| | | | | | hypothetical protein SSSM7_233 [*Synechococcus* phage S-SSM7] | | 4.47E-12 | 42.6% | |
| 230 | + | 195527 | 198295 | 922 | virion structural protein [uncultured Mediterranean phage uvMED] | concanavalin A-like lectin/glucanase | 4.00E-83 | 45% | pfam13385 (1.37e-05) |
| | | | | | virion structural protein [Cyanophage S-TIM5] | | 1.48E-79 | 36% | |
| 231 | + | 198356 | 200704 | 782 | long tail fiber proximal subunit [uncultured Mediterranean phage uvMED] | long tail fiber proximal subunit | 2.00E-162 | 55% | PRK15319 (5.14E-13) |
| | | | | | putative T4-like proximal tail fiber [*Prochlorococcus* phage P-SSM2] | | 1.15E-24 | 38.4% | |

| | | | | | | | | | |
|---|---|---|---|---|---|---|---|---|---|
| 232 | + | 200708 | 202678 | 656 | hypothetical protein [uncultured Mediterranean phage uvMED] | | 6.00E-78 | 34% | |
| | | | | | hypothetical protein Syn7803US120_166 [*Synechococcus* phage ACG-2014i] | | 5.36E-18 | 27.1% | |
| 234 | + | 202938 | 204593 | 551 | hypothetical protein CBE03_04315 [Gammaproteobacteria bacterium TMED243] | | 5.00E-19 | 58% | |
| | | | | | hypothetical protein Syn7803US120_166 [*Synechococcus* phage ACG-2014i] | | 5.36E-18 | 27.1% | |
| 235 | + | 204593 | 205699 | 368 | alpha/beta hydrolase fold [*Flavobacterium fontis*] | | 3.00E-24 | 36% | |
| 236 | + | 205762 | 208839 | 1025 | outer membrane protein [uncultured Mediterranean phage uvMED] | | 5.00E-33 | 36% | |
| 237 | + | 208951 | 209115 | 54 | hypothetical protein [uncultured Mediterranean phage uvMED] | | 2.00E-14 | 59% | |
| | | | | | hypothetical protein SSM2_101 [*Synechococcus* phage S-SM2] | | 2.02E-13 | 55.6% | |
| 238 | + | 209244 | 210446 | 400 | helicase [*Synechococcus* phage Bellamy] | helicase | 0 | 67% | pfam00270 (1.73E-05) |
| 240 | + | 210680 | 211348 | 222 | hypothetical protein N161109_037 [*Synechococcus* phage S-CAM9] | 2OG-Fe (II) oxygenase superfamily protein | 9.00E-40 | 43% | pfam13759 (2.20E-06) |
| 242 | + | 211901 | 212632 | 243 | hypothetical protein CBC18_00625 [Deltaproteobacteria bacterium TMED58] | | 6.00E-35 | 38% | |
| | | | | | hypothetical protein N161109_166 [*Synechococcus* phage S-CAM9] | | 1.43E-25 | 34.3% | |
| 243 | + | 212632 | 213294 | 220 | hypothetical protein [*Synechococcus* sp. CPC35] | 2OG-Fe (II) oxygenase superfamily protein | 8.00E-16 | 33.9% | pfam13759 (1.10E-13) |

| | | | | | | | | | |
|---|---|---|---|---|---|---|---|---|---|
| | | | | | phytanoyl-CoA-dioxygenase [*Prochlorococcus* phage P-SSM2] | | 2.76E-12 | 30.7% | |
| 244 | + | 213332 | 213868 | 178 | hypothetical protein [*Bradyrhizobium japonicum*] | | 2.00E-09 | 38% | |
| 245 | + | 213879 | 215144 | 421 | hypothetical protein CVU64_24165 [Deltaproteobacteria bacterium HGW-Deltaproteobacteria-21] | | 7.00E-12 | 45% | |
| 246 | + | 215181 | 215567 | 128 | hypothetical protein CBC18_00165 [Deltaproteobacteria bacterium TMED58] | | 3.00E-30 | 65% | |
| | | | | | hypothetical protein PSSM2_029 [*Prochlorococcus* phage P-SSM2] | | 2.12E-28 | 54.2% | |
| 248 | + | 216908 | 218269 | 453 | hypothetical protein CPRG_00149 [Cyanophage Syn30] | 2OG-Fe (II) oxygenase superfamily protein | 2.00E-69 | 57% | pfam13640 (1.39E-12) |
| | | | | | Ferrochelatase [*Synechococcus* phage S-PM2] | | 3.00E-59 | 50% | |
| 249 | + | 218266 | 218919 | 217 | 2OG-Fe(II) oxygenase [*Synechococcus* virus S-PRM1] | 2OG-Fe (II) oxygenase superfamily protein | 3.00E-57 | 46.7% | pfam13640 (9.21-14) |
| 250 | + | 218919 | 219134 | 71 | hypothetical protein SSSM7_309 [*Synechococcus* phage S-SSM7] | | 4.00E-10 | 41% | |
| | | | | | hypothetical protein CBB96_01765 [Gammaproteobacteria bacterium TMED36] | | 1.30E-09 | 40.9% | |
| 251 | + | 219115 | 219702 | 195 | hypothetical protein CBC18_03195 [Deltaproteobacteria bacterium TMED58] | 2OG-Fe (II) oxygenase superfamily protein | 1.00E-33 | 38% | pfam13640 (1.52-09) |
| | | | | | hypothetical protein Syn7803C100_23 [*Synechococcus* phage ACG-2014b] | | 8.00E-25 | 34.6% | |

| | | | | | | | | | |
|---|---|---|---|---|---|---|---|---|---|
| 252 | + | 219771 | 220139 | 122 | hypothetical protein PBI_BELLAMY_90 [*Synechococcus* phage Bellamy] | | 1.00E-62 | 77% | |
| | | | | | hypothetical protein SWZG_00210 [*Synechococcus* phage S-SKS1] | | 1.44E-39 | 55.4% | |
| 254 | + | 220303 | 220491 | 62 | hypothetical protein [Cyanophage S-TIM5] | | 5.00E-10 | 54% | |
| 256 | + | 220686 | 221237 | 183 | hypothetical protein SWPG_00188 [*Synechococcus* phage S-CBM2] | | 5.00E-84 | 84% | |
| 257 | + | 221406 | 222290 | 294 | hypothetical protein [uncultured Mediterranean phage uvMED] | | 3.00E-73 | 43% | |
| | | | | | hypothetical protein S420910_059 [*Synechococcus* phage S-CAM7] | | 8.71E-69 | 41.7% | |
| 258 | + | 222361 | 222546 | 61 | hypothetical protein [*Synechococcus* phage S-PM2] | | 4.00E-05 | 45% | |
| 259 | + | 222584 | 223018 | 144 | hypothetical protein [uncultured Mediterranean phage uvMED] | | 3.00E-51 | 56% | |
| | | | | | hypothetical protein SSM2_057 [*Synechococcus* phage S-SM2] | | 1.63E-43 | 51.4% | |
| 260 | + | 223025 | 223330 | 101 | hypothetical protein SSM2_054 [S*ynechococcus* phage S-SM2] | | 5.00E-10 | 36% | |
| 261 | + | 223330 | 223716 | 128 | dCMP deaminase [*Synechococcus* phage ACG-2014f] | dCMP deaminase (T4-like) | 9.00E-52 | 59% | pfam15112 (8.00E-04) |
| 263 | + | 224134 | 224373 | 79 | hypothetical protein CBC89_04890 [*Euryarchaeota archaeon* TMED129] | | 3.00E-17 | 48% | |
| 264 | + | 224373 | 224705 | 110 | hypothetical protein [uncultured Mediterranean phage uvMED] | | 2.00E-38 | 69% | |

| | | | | | | | |
|---|---|---|---|---|---|---|---|
| | | | | | hypothetical protein PBI_BELLAMY_100 [*Synechococcus* phage Bellamy] | 1.17E-44 | 64.2% |
| 266 | + | 224938 | 225051 | 37 | hypothetical protein SWSG_00013 [*Synechococcus* phage S-RIM8 A.HR5] | 1.00E-08 | 64% |
| 267 | + | 225048 | 225236 | 62 | gp64 [*Synechococcus* phage syn9] | 2.00E-23 | 68% |
| 268 | + | 225236 | 225514 | 92 | hypothetical protein Syn9311C1_66 [*Synechococcus* phage ACG-2014b] | 6.00E-43 | 92% |
| 269 | + | 225514 | 225714 | 66 | hypothetical protein [uncultured Mediterranean phage uvMED] | 1.00E-34 | 83% |
| | | | | | hypothetical protein R290704_068 [Cyanophage S-RIM50] | 1.07E-13 | 48.5% |
| 270 | + | 225711 | 225878 | 55 | hypothetical protein SSSM5_067 [*Synechococcus* phage S-SSM5] | 1.00E-18 | 69% |
| 271 | + | 225881 | 226099 | 72 | hypothetical protein Syn7803US23_50 [*Synechococcus* phage ACG-2014j] | 4.00E-31 | 79% |
| 272 | + | 226096 | 226371 | 91 | hypothetical protein SXBG_00054 [*Synechococcus* phage S-CAM1] | 3.00E-45 | 76% |
| 273 | + | 226472 | 226708 | 78 | hypothetical protein T191209_062 [*Synechococcus* phage S-CAM22] | 3.00E-45 | 91% |
| 274 | + | 226742 | 226906 | 54 | hypothetical protein SWYG_00040 [*Synechococcus* phage S-IOM18] | 4.00E-25 | 83% |
| 275 | + | 226909 | 227040 | 43 | hypothetical protein Syn19_075 [*Synechococcus* phage Syn19] | 2.00E-15 | 80% |
| 277 | + | 227196 | 227441 | 81 | hypothetical protein [uncultured Mediterranean phage uvMED] | 4.00E-23 | 73% |

| | | | | | | | |
|---|---|---|---|---|---|---|---|
| | | | | | gp82 [*Synechococcus* phage syn9] | 3.00E-05 | 45% |
| 278 | + | 227456 | 227662 | 68 | hypothetical protein Syn1_066 [*Prochlorococcus* phage Syn1] | 6.00E-16 | 56% |
| 279 | + | 227782 | 227922 | 46 | hypothetical protein N330309_171 [*Synechococcus* phage S-CAM1] | 2.00E-20 | 89% |
| 282 | + | 228259 | 228567 | 102 | hypothetical protein Syn33_067 [*Prochlorococcus* phage Syn33] | 2.00E-27 | 60% |

[a]The top homolog and additional homologs for each organism type that is different from the top one are displayed based on BLASTP against the non-redundant database.

[b]Putative functions are predicted based on the function of homologs from the Conserved Domain Database.

[c]The best hit of each ORF in the Conserved Domain Database and its e-value.

Table S2. Predicted ORFs in the S-SCSM1 genome that have distant homologs detected by using HHpred and Phyre2 search.

| ORF | Strand | Left | Right | aa length | Putative function[a] | HHpred search | | | Phyre2 search | | |
|---|---|---|---|---|---|---|---|---|---|---|---|
| | | | | | | Hits | Probability | E-value | Hits | Confidence | Identity |
| 1 | + | 1 | 531 | 176 | 2OG-Fe (II) oxygenase superfamily protein | prolyl-4 hydroxylase [*Chlamydomonas reinhardtii*] | 99.8% | 4.4E-18 | PKHD-type hydroxylase TPA1 [*Saccharomyces cerevisiae*] | 99.6% | 20% |
| 16 | + | 20510 | 21061 | 183 | 2OG-Fe (II) oxygenase superfamily protein | PKHD-type hydroxylase [*Shewanella baltica* OS155] | 99.2% | 2.5E-09 | oxoglutarate-iron-dependent dioxygenase [*Homo sapiens*] | 96.4% | 14% |
| 17 | + | 21084 | 21863 | 259 | baseplate wedge protein gp10 | the trigger of tail contraction and the long tail fibers connector [Bacteriophage T4] | 98.7% | 1.5E-08 | baseplate wedge protein gp10 [*Enterobacteria* virus T4] | 97.3% | 50% |
| | | | | | | baseplate wedge protein gp10 [*Enterobacteria* phage T4] | 98.2% | 1.8E-06 | | | |
| 20 | + | 31275 | 31493 | 72 | prefoldin | prefoldin [*Methanothermobacter thermautotrophicus*] | 94.5% | 1.2 | no hit | | |
| 21 | + | 31475 | 31861 | 128 | tail fiber protein | L-shaped tail fiber protein p132 [*Escherichia* phage T5] | 99.9% | 6.7E-21 | no hit | | |
| 26 | + | 35707 | 40143 | 1478 | long tail fiber protein | long tail fiber protein p37 [*Escherichia* phage AR1] | 98.1% | 5.8E-06 | pectinesterase A [*Dickeya chrysanthemi*] | 93.8% | 17% |
| | | | | | | glycoside hydrolase BT_1002 [*Bacteroides thetaiotaomicron*] | 96.6% | 3.2E-03 | | | |
| 33 | + | 46947 | 47168 | 73 | endonuclease | putative nuclease [*Acanthamoeba polyphaga* mimivirus (APMV)] | 98.1% | 6.0E-06 | no hit | | |
| | | | | | | restriction endonuclease Hpy99I [*Helicobacter pylori* J99] | 97.5% | 1.1E-04 | | | |
| 48 | + | 59192 | 59581 | 129 | Sm-like RNA-binding | Sm-like RNA-binding protein [putative | 99.9% | 1.7E-21 | Sm-like RNA-binding protein | 96.1% | 29% |

| | | | | | | | | | | | |
|---|---|---|---|---|---|---|---|---|---|---|---|
| | | | | | protein | cyanophage] | | | [putative cyanophage] | | |
| 51 | + | 61233 | 61502 | 89 | Tic22-like family protein | Tic22-like family chaperon [*Anabaena* sp.] | 96.1% | 1.9E-01 | no hit | | |
| 54 | + | 64060 | 64380 | 106 | endonuclease | type II intron maturase [*Lactococcus lactis*] | 98.6% | 1.0E-07 | HNH endonuclease [*Escherichia coli* K-12] | 99.4% | 27% |
| | | | | | | nicking endonuclease [*Escherichia* phage T5] | 98.5% | 3.6E-07 | | | |
| 55 | + | 64405 | 66540 | 711 | midasin | midasin/rRNA-processing protein [*Saccharomyces cerevisiae*] | 99.2% | 3.7E-10 | integrin beta-1 [*Homo sapiens*] | 98% | 19% |
| 61 | + | 69658 | 70263 | 201 | 2OG-Fe (II) oxygenase superfamily protein | proly-4-hydroxylase [*Chlamydomonas reinhardtii*] | 99.6% | 2.1E-13 | 2OG-Fe(II) oxygenase superfamily [*Homo sapiens*] | 100% | 19% |
| 71 | + | 74989 | 75564 | 191 | 2OG-Fe (II) oxygenase superfamily protein | putative 2OG-Fe (II) oxygenase [*Oceanicola granulosus* HTCC2516] | 100% | 2.7E-29 | no hit | | |
| 84 | + | 84631 | 85167 | 178 | 2OG-Fe (II) oxygenase superfamily protein | proly-4-hydroxylase [*Chlamydomonas reinhardtii*] | 99.8% | 1.6E-16 | prolyl 4-hydroxylase subunit alpha [*Dictyostelium discoideum*] | 99.9% | 20% |
| 95 | + | 92158 | 92691 | 177 | tail fiber protein | tail fiber protein [*Escherichia* phage T1] | 97.2% | 2.2E-03 | no hit | | |
| 98 | + | 93281 | 93712 | 143 | holin-like protein | holin-like protein [*Bacillus* phage SPP1] | 98% | 8.7E-04 | no hit | | |
| 111 | + | 101893 | 102069 | 58 | YueI-like protein | no hit | | | YueI-like [*Bacillus subtilis*] | 81% | 53% |
| 114 | + | 103925 | 104620 | 231 | 2OG-Fe(II) oxygenase superfamily protein | oxygenase [*Streptomyces viridochromogenes* Tue57] | 99.2% | 3.2E-09 | oxygenase [*Streptomyces viridochromogenes* Tue57] | 97% | 16% |
| 124 | + | 111285 | 112136 | 283 | coatomer subunit alpha | coatomer subunit alpha [*Schizosaccharomyces pombe*] | 99.8% | 7.9E-15 | no hit | | |
| | | | | | | surface layer protein [*Methanosarcina mazei*] | 99.8% | 4.5E-14 | | | |

| | | | | | | | | | | | |
|---|---|---|---|---|---|---|---|---|---|---|---|
| 125 | + | 112133 | 113002 | 289 | DNA beta-glucosyltransferase | DNA beta-glucosyltransferase [*Enterobacteria* phage T4] | 99.9% | 3.0E-24 | no hit | | |
| 129 | + | 115338 | 116030 | 230 | glycosytransferase | glycosyltransferase family 8 [*Anaerococcus prevotii*] | 99.3% | 3.4E-11 | WbbM bifunctional glycosyltransferase [*Klebsiella pneumoniae*] | 97.7% | 13% |
| | | | | | | galactosyltransferase LgtC [*Neisseria meningitidis*] | 99.2% | 1.8E-10 | galactosyltransferase Lgtc [*Neisseria meningitidis*] | 96.6% | 11% |
| 132 | + | 117900 | 118700 | 266 | glycosytransferase | galactosyltransferase LgtC [*Neisseria meningitidis*] | 99.9% | 7.5E-20 | WbbM bifunctional glycosyltransferase [*Klebsiella pneumoniae*] | 96.8% | 20% |
| 134 | + | 119271 | 120062 | 263 | fucosyltransferase NodZ | fucosyltransferase NodZ [*Bradyrhizobium* sp.] | 100% | 3.3E-27 | fucosyltransferase NodZ [*Bradyrhizobium* sp.] | 98.7% | 19% |
| 138 | + | 121501 | 121662 | 53 | AbrB family transcriptional regulator | AbrB family transcriptional regulator [*Sulfolobus solfataricus*] | 91.4% | 1.5 | no hit | | |
| 141 | + | 122561 | 123166 | 201 | morphogenesis protein 1 | morphogenesis protein 1 [*Bacillus* phage B103] | 96.3% | 5.5E-01 | morphogenesis protein 1 (tail lysozyme) [*Bacillus* virus phi29] | 100% | 21% |
| 143 | + | 123687 | 124058 | 123 | outer membrane autotransporter barrel | outer membrane protein lom [*Escherichia* phage lambda] | 98.9% | 1.4E-06 | outer membrane autotransporter barrel [*Escherichia coli*] | 97.3% | 15% |
| 144 | + | 124256 | 124615 | 119 | outer membrane autotransporter barrel | outer membrane protein A [*Escherichia coli*] | 98.6% | 1.6E-05 | outer membrane autotransporter barrel [*Escherichia coli*] | 95.8% | 14% |
| 146 | + | 125610 | 125876 | 88 | cell cycle protein | initiation-control protein YabA, replication [*Bacillus subtilis* st. 168] | 93.5% | 1.2 | no hit | | |
| | | | | | | cell division protein FtsQ [*Escherichia coli* K-12] | 92.2% | 1 | | | |
| 148 | + | 126146 | 126982 | 278 | proteolytic enzyme | proteolytic enzyme [*Methanocaldococcus jannaschii*] | 95.7% | 9.7E-02 | no hit | | |

| | | | | | | | | | | | |
|---|---|---|---|---|---|---|---|---|---|---|---|
| 162 | + | 132842 | 133618 | 258 | arginase-like amidino hydrolase | 3-guanidinopropionase [*Pseudomonas aeruginosa* PA01] | 97% | 1.0E-02 | no hit | | |
| | | | | | | agmatinase [*Deinococcus radiodurans*] | 96.7% | 2.2E-02 | | | |
| 170 | + | 139635 | 140225 | 196 | 2OG-Fe (II) oxygenase superfamily protein | prolyl 4-hydroxylase [*Chlamydomonas reinhardtii*] | 99.6% | 1.0E-14 | prolyl 4-hydroxylase [*Pseudomonas putida* KT2440] | 98.8% | 13% |
| 180 | + | 143530 | 144027 | 165 | DNA ligase | DNA ligase [*Enterobacteria* phage T4] | 99.9% | 7.7E-25 | no hit | | |
| 186 | + | 145303 | 145917 | 204 | 2OG-Fe (II) oxygenase superfamily protein | oxygenase [*Streptomyces viridochromogenes* Tue57] | 99.9% | 6.0E-23 | no hit | | |
| 187 | + | 145892 | 146131 | 79 | thioredoxin | thioredoxin [*Mus musculus*] | 99.3% | 1.1E-09 | no hit | | |
| 192 | + | 147104 | 147517 | 137 | chromosome segregation protein | chromosome segregation [*Geobacillus thermoleovorans* CCB_US3_UF5] | 97.8% | 5.0E-05 | no hit | | |
| 199 | + | 152825 | 153076 | 83 | transcription factor RbpA (T4-like gp39) | transcription factor RbpA [*Streptomyces coelicolor*] | 98.2% | 1.2E-05 | no hit | | |
| | | | | | | RBP12 subunit of RNA polymerase II [*Saccharomyces cerevisiae*] | 98.1% | 1.3E-05 | | | |
| 208 | - | 159832 | 160179 | 115 | terminal DNA protecting protein | terminal DNA protecting protein [*Enterobacteria* phage T4] | 100% | 1.1E-30 | no hit | | |
| 210 | + | 160668 | 161522 | 284 | tail tube protein (T4-like gp19) | tail tube protein gp19 [*Serratia* phage KSP90] | 99.9% | 4.2E-24 | tail tube protein [*Enterobacteria* phage T4 sensu lato] | 99.7% | 19% |
| 215 | + | 168714 | 170018 | 434 | baseplate hub protein (T4-like gp27) | baseplate lysozyme, hub [*Enterobacteria* phage T4] | 100% | 9.9E-37 | baseplate assembly protein [*Shewanella oneidensis* MR-1] | 98.9% | 11% |
| 217 | + | 170258 | 173017 | 919 | pre-baseplate central spike protein (T4-like gp5) | pre-baseplate central spike protein gp5 [*Escherichia* virus T4] | 99.3% | 6.0E-12 | baseplate central spike complex protein gp27 [*Escherichia* virus T4] | 99.9% | 25% |

| | | | | | | | | | | | |
|---|---|---|---|---|---|---|---|---|---|---|---|
| | | | | | | tail-associated lysozyme gp5 [Bacteriophage T4] | 98.9% | 8.5E-09 | pre-baseplate central spike protein gp5 [*Escherichia* virus T4] | 99.4% | 25% |
| 218 | + | 173044 | 173628 | 194 | contractile injection system tube protein | contractile injection system tube protein [*Serratia entomophila*] | 95.4% | 4.8E-01 | no hit | | |
| 223 | + | 175821 | 175985 | 54 | Rob transcription factor | Rob transcription factor [*Escherichia coli*] | 96.1% | 9.5E-03 | no hit | | |
| 225 | + | 176745 | 182306 | 1853 | tail fiber protein | endosialidase [*Enterobacteria* phage phi92] | 90.5% | 1.3 | rhamnogalacturonan lyase [*Bacteroides thetaiotaomicron*] | 97% | 22% |
| | | | | | | | | | pectin lyase-like protein [*Dickeya chrysanthemi*] | 96.3% | 18% |
| 226 | + | 182327 | 186364 | 1345 | outer capsid protein | hoc head outer capsid protein [*Escherichia* phage RB49] | 97.8% | 1.0E-03 | no hit | | |
| | | | | | | outer capsid protein [*Escherichia* virus T4] | 97.5% | 5.2E-02 | | | |
| 235 | + | 204593 | 205699 | 368 | tail fiber protein | alpha/beta-hydrolases/aminopeptidase [*Streptomyces morookaense*] | 99.8% | 2.9E-17 | alpha/beta-hydrolases/oxidized polyvinyl alcohol hydrolase [*Sphingopyxis* sp. 113P3] | 89.7% | 24% |
| 237 | + | 208951 | 209115 | 54 | phycobilisome degradation protein NblA | phycobilisome degradation protein NblA [*Nostoc* sp. PCC 7120] | 95.5% | 2.4E-01 | no hit | | |
| | | | | | | phycobilisome degradation protein NblA [*Synechococcus elongatus* PCC 7942] | 95% | 2.7E-01 | | | |
| 241 | + | 211341 | 211904 | 187 | 2OG-Fe(II) oxygenase superfamily protein | prolyl-4 hydroxylase [*Chlamydomonas reinhardtii*] | 99.5% | 4.6E-12 | Fe-2OG dioxygenase domain-containing protein [*Pseudomonas putida* KT2440] | 87.8% | 14% |
| 242 | + | 211901 | 212632 | 243 | 2OG-Fe(II) oxygenase superfamily protein | 2OG-Fe (II) oxygenase domain-containing protein [*Homo | 98.5% | 5.0E-06 | 2-oxoglutarate and iron-dependent oxygenase [*Homo sapiens*] | 84.7% | 17% |

| | | | | | | | | | | | |
|---|---|---|---|---|---|---|---|---|---|---|---|
| | | | | | | *sapiens*] | | | | | |
| 244 | + | 213332 | 213868 | 178 | tail fiber protein | baseplate structural protein gp10 [*Enterobacteria* phage T4] | 98.9% | 3.6E-08 | long tail fiber protein gp37 [*Escherichia* virus T4] | 99.5% | 19% |
| | | | | | | | | | short tail fiber protein gp12 [*Escherichia* virus T4] | 99.3% | 10% |
| | | | | | | | | | baseplate wedge protein gp10 [*Escherichia* virus T4] | 95.8% | 16% |
| 245 | + | 213879 | 215144 | 421 | short tail fiber protein (T4-like gp12) | short tail fibre protein gp12 [*Enterobacteria* phage T4] | 99.2% | 6.1E-11 | no hit | | |
| 264 | + | 224373 | 224705 | 110 | transcriptional regulator of DNA damage repair IrrE | transcriptional regulator of DNA damage repair IrrE [*Deinococcus deserti*] | 98.7% | 3.8E-07 | no hit | | |
| 279 | + | 227782 | 227922 | 46 | RNA polymerase inhibitor | RNA polymerase inhibitor [*Enterobacteria* phage T7] | 92.9% | 2.3 | no hit | | |

[a]Putative functions are predicted based on the function of homologs using HHpred and Phyre2 search.

Table S3. Amino acid sequence identities among the SCSM1 2OG-Fe(II) oxygenase family proteins.

| | ORF63 | ORF71 | ORF77 | ORF79 | ORF80 | ORF107 | ORF120 | ORF240 | ORF243 | ORF16 | ORF170 | ORF88 | ORF249 | ORF112 | ORF224 | ORF248 | ORF251 | ORF1 | ORF61 | ORF84 | ORF241 | ORF242 | ORF114 | ORF186 |
|---|---|---|---|---|---|---|---|---|---|---|---|---|---|---|---|---|---|---|---|---|---|---|---|---|
| ORF63 | 100 | - | - | - | - | - | - | - | - | - | - | - | - | - | - | - | - | - | - | - | - | - | - | - |
| ORF71 | | 100 | - | - | - | - | - | 25.24 | - | - | - | - | | - | - | - | - | - | - | - | - | - | - | - |
| ORF77 | | | 100 | - | - | - | - | - | - | - | - | - | - | - | - | - | - | - | - | - | - | - | - | - |
| ORF79 | | | | 100 | 44.16 | - | - | - | 26.96 | - | - | - | - | - | - | - | - | - | - | - | - | - | - | - |
| ORF80 | | | | | 100 | - | 26.15 | - | 27.15 | - | - | - | - | - | - | - | - | - | - | - | - | - | - | - |
| ORF107 | | | | | | 100 | - | - | - | - | - | - | - | - | - | - | - | - | - | - | - | - | - | - |
| ORF120 | | | | | | | 100 | - | - | - | - | - | - | - | - | - | - | - | - | - | - | - | - | - |
| ORF240 | | | | | | | | 100 | 43.14 | - | - | - | - | - | - | - | - | - | - | - | - | - | - | - |
| ORF243 | | | | | | | | | 100 | - | - | - | - | - | - | - | - | - | - | - | - | - | - | - |
| ORF16 | | | | | | | | | | 100 | - | - | - | - | - | - | - | - | - | - | - | - | - | - |
| ORF170 | | | | | | | | | | | 100 | - | - | - | - | - | - | - | - | - | - | - | | - |
| ORF88 | | | | | | | | | | | | 100 | 38.41 | - | - | - | - | - | - | - | - | - | - | - |
| ORF249 | | | | | | | | | | | | | 100 | - | - | - | - | - | - | - | - | - | - | - |
| ORF112 | | | | | | | | | | | | | | 100 | 37.57 | 31.13 | - | - | - | - | - | - | - | - |
| ORF224 | | | | | | | | | | | | | | | 100 | 26.34 | - | - | - | - | - | - | - | - |
| ORF248 | | | | | | | | | | | | | | | | 100 | - | - | - | - | - | - | - | - |
| ORF251 | | | | | | | | | | | | | | | | | 100 | - | - | - | - | - | - | - |
| ORF1 | | | | | | | | | | | | | | | | | | 100 | 25 | 24.86 | - | - | - | - |
| ORF61 | | | | | | | | | | | | | | | | | | | 100 | 23.53 | - | - | - | - |
| ORF84 | | | | | | | | | | | | | | | | | | | | 100 | - | - | - | - |
| ORF241 | | | | | | | | | | | | | | | | | | | | | 100 | - | - | - |
| ORF242 | | | | | | | | | | | | | | | | | | | | | | 100 | - | - |
| ORF114 | | | | | | | | | | | | | | | | | | | | | | | 100 | - |
| ORF186 | | | | | | | | | | | | | | | | | | | | | | | | 100 |

Table S4. The S-SCSM1 *nblA* homologs from cyanomyoviruses are distant homology with cyanobacterial *nblAs* detected by HHpred search.

| Cyanophage | Protein ID in NR database | Morphotype | Habitat | Best hit in the HHpred search | Probility | E-value |
|---|---|---|---|---|---|---|
| *Synechococcus* phage S-B43 | QDH50654.1 | Myovirus | marine | NblA protein [*Thermostichus vulcanus*] | 96.6% | 4.8E-02 |
| *Synechococcus* phage S-B05 | QCW23002.1 | Myovirus | marine | NblA protein [*Thermostichus vulcanus*] | 96.5% | 5.7E-02 |
| *Synechococcus* phage S-SM2 | YP_004322257.1 | Myovirus | marine | NblA protein [*Thermostichus vulcanus*] | 96.8% | 3.0E-02 |
| *Synechococcus* phage Bellamy | YP_009791272.1 | Myovirus | marine | NblA protein [*Thermostichus vulcanus*] | 97.3% | 7.4E-03 |
| *Synechococcus* phage S-SKS1 | YP_007674584.1 | Myovirus | marine | NblA protein [*Thermostichus vulcanus*] | 96.4% | 6.3E-02 |
| *Synechococcus* phage SynMITS9220M01 | QJT70100.1 | Myovirus | marine | NblA protein [*Thermostichus vulcanus*] | 97.2% | 7.6E-03 |
| *Synechococcus* phage S-SRM01 | QPX48163.1 | Myovirus | freshwater | NblA protein [*Thermostichus vulcanus*] | 96.9% | 2.5E-02 |

Table S5. *Cis*-regulatory RNAs found in cyanophages.*

| Cyanophage | Feature | Start | End | Score |
|---|---|---|---|---|
| S-SCSM1 | *wcaG* | 107200 | 107286 | 37.11 |
| | PhotoRC-II | 137169 | 137262 | 75.99 |
| *Synechococcus* phage S-SM2 | *wcaG* | 143,437 | 143,532 | 91.6 |
| | PhotoRC-II | 179,573 | 179,668 | 69.8 |
| *Synechococcus* phage S-RSM4 | PhotoRC-II RNA | 8,698 | 8,606 | 62.3 |
| | *wcaG* RNA | 29,718 | 29,624 | 89.4 |
| *Synechococcus* phage syn9 | ncRNA | 163,184 | 163,278 | 77.9 |
| | PhotoRC-II | 165,875 | 165,970 | 62.8 |
| *Synechococcus* phage S-PM2 | ncRNA | 122,806 | 122,905 | 70.8 |
| *Synechococcus* phage ACG-2014j | *wcaG* | 161,256 | 161,354 | 63.1 |
| | PhotoRC-II | 180,801 | 180,896 | 73.8 |
| *Synechococcus* phage S-RIM8 A.HR1 | *wcaG* | 157,556 | 157,661 | 62.4 |
| | PhotoRC-II | 160,699 | 160,790 | 58.5 |
| *Synechococcus* phage ACG-2014e | *wcaG* | 156,760 | 156,858 | 58.7 |
| | PhotoRC-II | 177,668 | 177,763 | 75.5 |
| *Synechococcus* phage S-RIM2 R1_1999 | *wcaG* | 162,743 | 162,837 | 58.2 |
| | PhotoRC-II | 165,583 | 165,678 | 74.1 |
| *Synechococcus* phage S-MbCM7 | *wcaG* | 155,033 | 155,127 | 49.5 |
| | *wcaG* | 155,137 | 155,239 | 43 |
| | PhotoRC-II | 177,377 | 177,473 | 63.4 |
| *Prochlorococcus* phage Syn1 | *wcaG* | 167,073 | 167,166 | 44 |
| | *wcaG* | 167,176 | 167,284 | 42.3 |
| | PhotoRC-II | 181,614 | 181,709 | 75.6 |
| *Synechococcus* phage S-MbCM7 | *wcaG* | 155,033 | 155,127 | 49.5 |
| | *wcaG* | 155,137 | 155,239 | 43 |
| | PhotoRC-II | 177,377 | 177,473 | 63.4 |
| *Prochlorococcus* phage P-RSM4 | *manA* | 120,783 | 120,973 | 144.3 |
| | PhotoRC-II | 168,301 | 168,400 | 77.4 |
| *Synechococcus* phage S-SM1 | PhotoRC-II | 163,608 | 163,699 | 71.6 |
| *Synechococcus* phage Syn19 | PhotoRC-II | 166,909 | 167,003 | 78.3 |
| *Prochlorococcus* phage Syn33 | PhotoRC-II | 162,206 | 162,301 | 74.2 |

* S-SCSM1 *cis*-regulatory RNAs were identified by searching against the Rfam database, whereas ncRNA information of other cyanophages were obtained from the Rfam database.

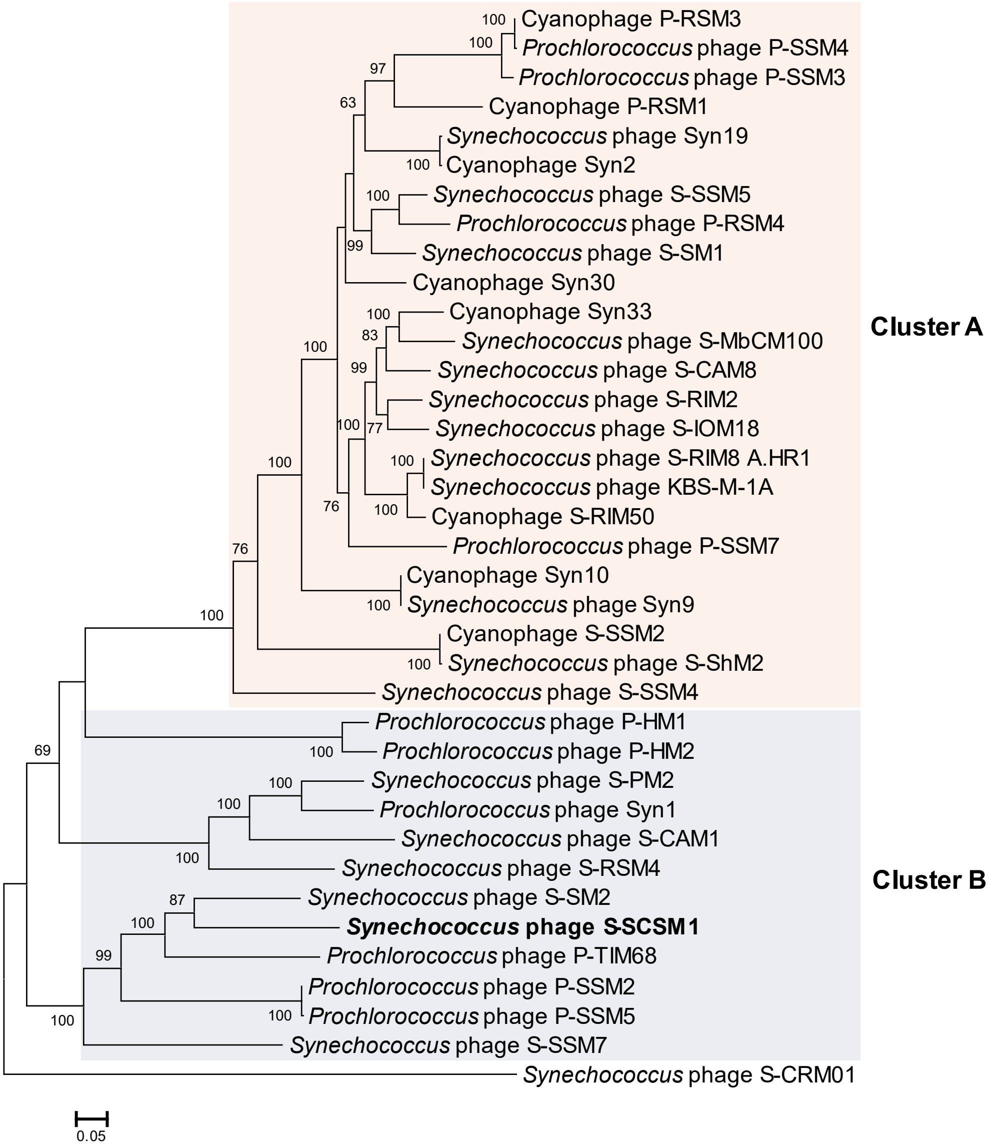


Figure S1. Phylogenomic analysis of S-SCSM1 and 36 T4-like cyanophages based on the concatenated alignments of 34 single-copy orthologous groups using the maximum-likelihood method. Bootstrap tests were performed with 100 replicates.

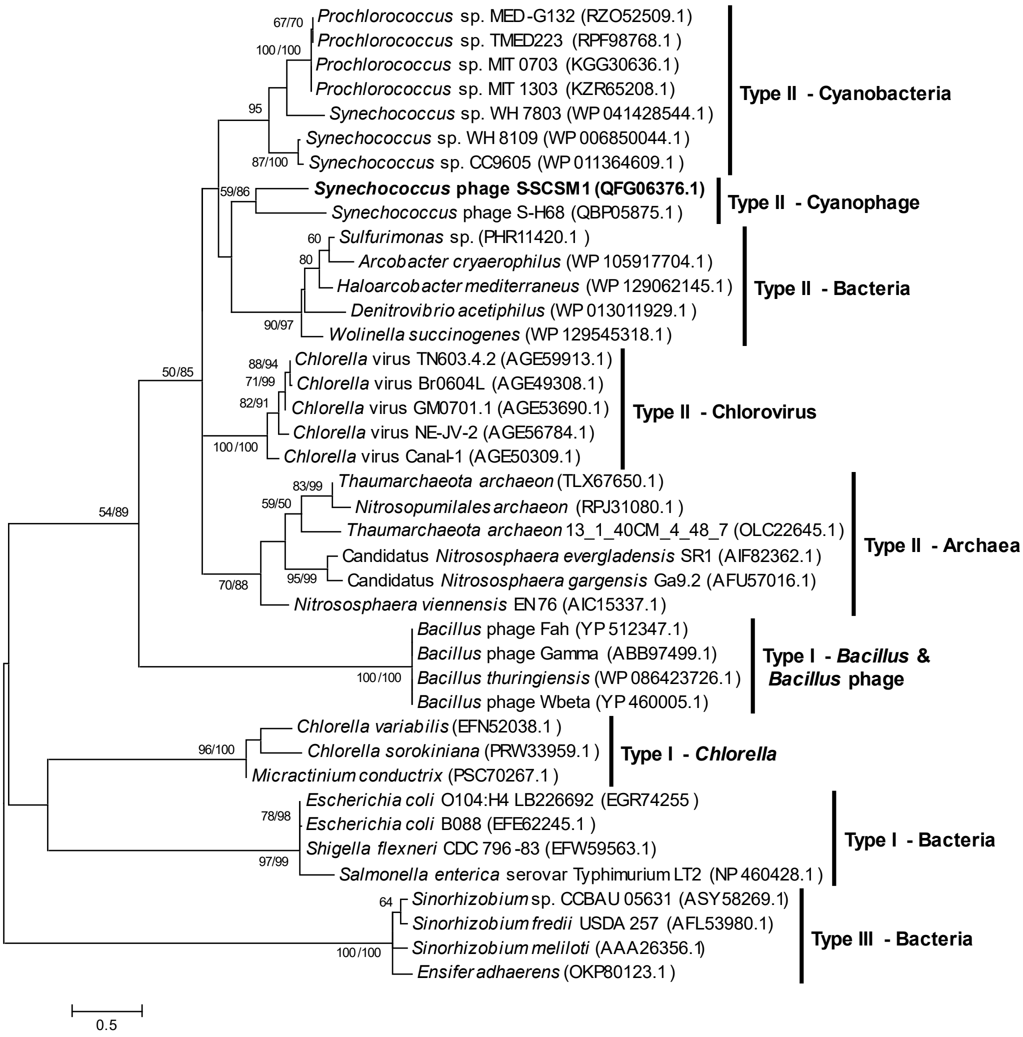


Figure S2. Unrooted Maximum-likelihood phylogenetic tree constructed using amino acid sequences of Type I, II, and III MPIs from cyanophages, *Bacillus* phages, Chlorovirus, cyanobacteria, bacteria, Archaea, and *Chlorella*. The bootstrap values (Maximum-likelihood/Neighbor-joining) of $\geq$ 50% are shown near each node. Number of bootstrap replicates = 1,000.

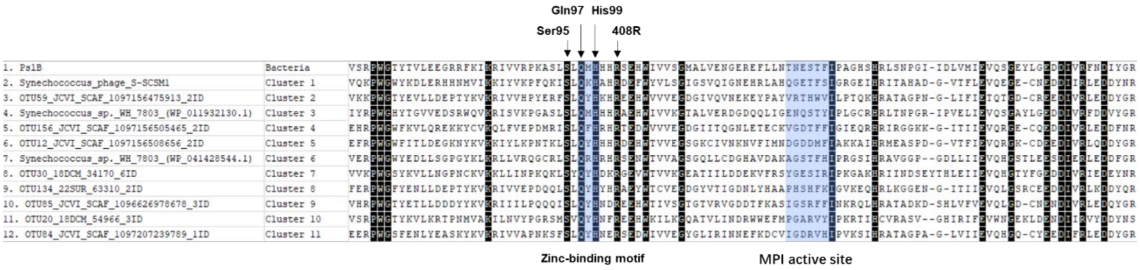


Figure S3. Alignment of representative cyanophage and picocyanobacterial MPI amino acid sequences from the 11 clusters. PslB from *Pseudomonas aeruginosa* PAO1 was included as a reference. The Zinc-binding motif and MPI active site are indicated by blue shadowing. The important amino acid residues are indicated by arrows.

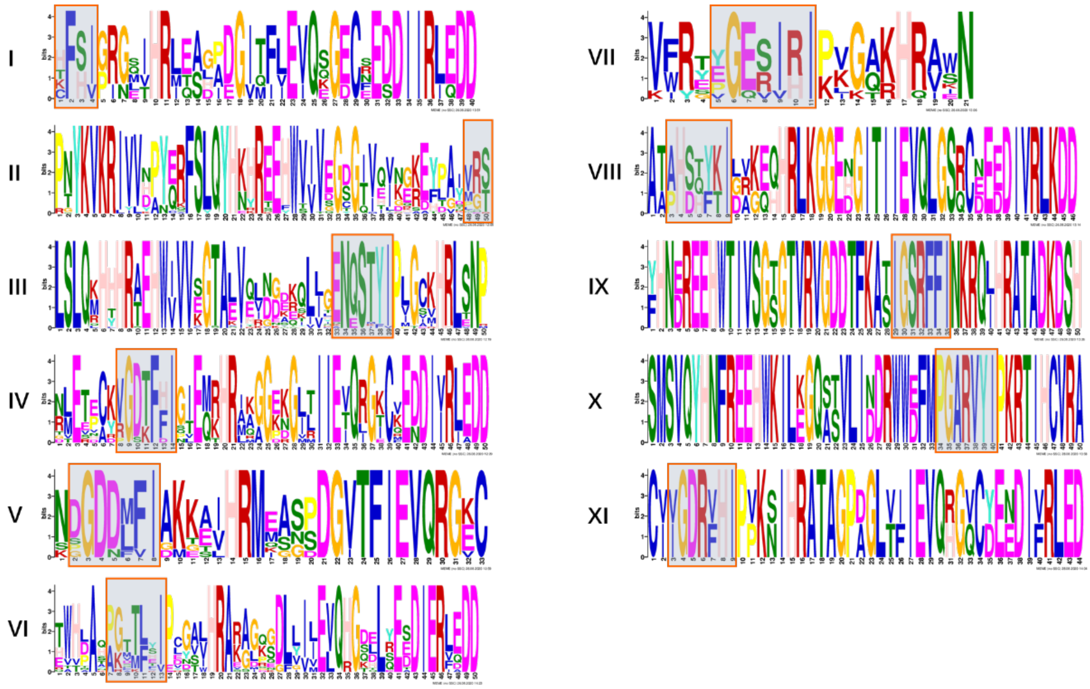


Figure S4. Motifs of the 11 cyanophage clusters containing the MPI active site. MPI active sites in the different clusters are indicated by orange boxes.

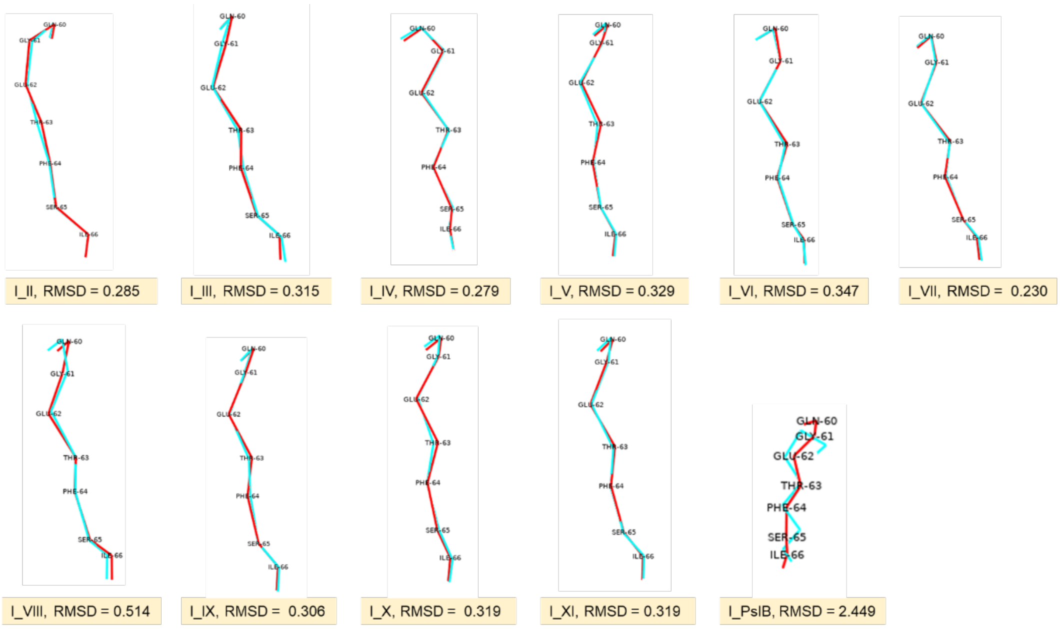


Figure S5. Comparisons between the predicted tertiary structure of Cluster I and those of the 10 other clusters identified in the phylogenetic analysis, as well as that of the reference protein *Pseudomonas aeruginosa* PslB. The predicted structure of the S-SCSM1 MPI active site is represented by a red line in each comparison, while the predicted structures of the MPI active sites in representative sequences from the other ten clusters and PslB in *Pseudomonas aeruginosa* PAO1 are represented by cyan lines.

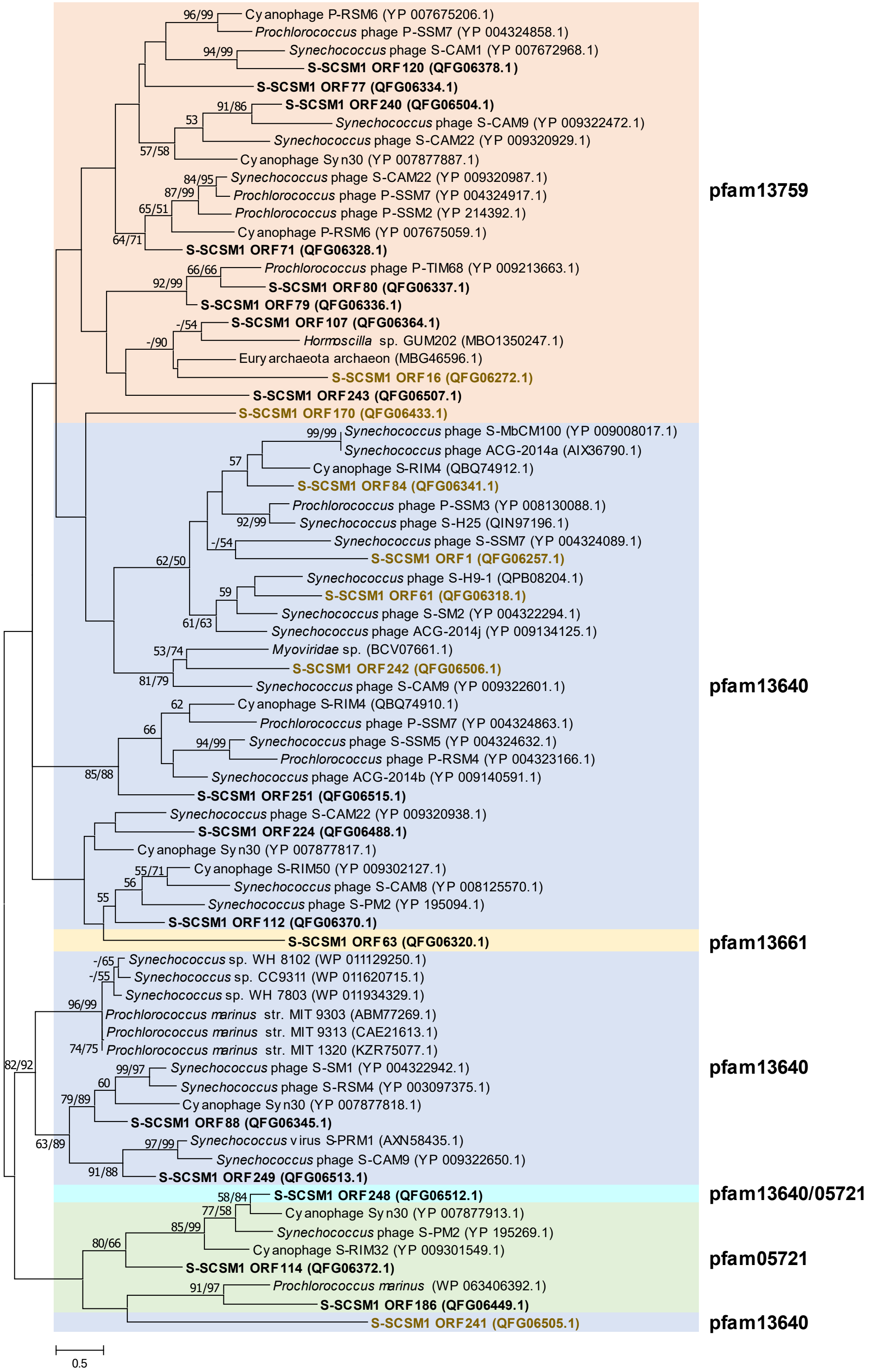


Figure S6. Phylogeny of the S-SCSM1 predicted 2OG-Fe(II) oxygenase superfamily proteins. An unrooted maximum-likelihood tree based on amino acid sequences is shown. ORFs containing the pfam13640, -13661, -13759, and -05721 conserved

domain were marked in blue, yellow, orange, and green shadows, respectively. ORF248 contains both the pfam13640 and -05721 conserved domains, and was marked in the cyan shadow. ORFs in brown font were identified based on predicted structural properties. Bootstrap values of ≥50% (maximum-likelihood/neighbor-joining) are shown near each node. Number of bootstrap replicates = 1,000.
